# Bulk and single-cell transcriptome profiling reveal necroptosis-based molecular classification, tumor microenvironment infiltration characterization, and prognosis prediction in colorectal cancer

**DOI:** 10.1186/s12967-022-03431-6

**Published:** 2022-05-19

**Authors:** Wenqin Luo, Wenqiang Xiang, Lu Gan, Ji Che, Jing Li, Yichao Wang, Lingyu Han, Ruiqi Gu, Li Ye, Renjie Wang, Xiuping Zhang, Ye Xu, Weixing Dai, Shaobo Mo, Qingguo Li, Guoxiang Cai

**Affiliations:** 1 Department of Colorectal Surgery, Fudan University Shanghai Cancer Center, Shanghai, China; 2 Department of Oncology, Shanghai Medical College, Fudan University, Shanghai, China; 3 Department of Medical Oncology, Zhongshan Hospital, Fudan University, Shanghai, China; 4 Department of CyberKnife Center, Huashan Hospital, Fudan University, Shanghai, China; 5 Department of Anesthesiology, Fudan University Shanghai Cancer Center, Shanghai, China; 6 Xiamen Clinical Research Center for Cancer Therapy, Xiamen Branch, Zhongshan Hospital, Fudan University, Xiamen, China; 7 Department of Cancer Center, Zhongshan Hospital, Fudan University, Shanghai, China; 8 Department of Center of Evidence-Based Medicine, Fudan University, Shanghai, China

**Keywords:** Colorectal cancer, Necroptosis, Tumor microenvironment, Immunotherapy, Drug sensitivity

## Abstract

**Background:**

Necroptosis is a new form of programmed cell death that is associated with cancer initiation, progression, immunity, and chemoresistance. However, the roles of necroptosis-related genes (NRGs) in colorectal cancer (CRC) have not been explored comprehensively.

**Methods:**

In this study, we obtained NRGs and performed consensus molecular subtyping by “ConsensusClusterPlus” to determine necroptosis-related subtypes in CRC bulk transcriptomic data. The ssGSEA and CIBERSORT algorithms were used to evaluate the relative infiltration levels of different cell types in the tumor microenvironment (TME). Single-cell transcriptomic analysis was performed to confirm classification related to NRGs. NRG_score was developed to predict patients’ survival outcomes with low-throughput validation in a patients’ cohort from Fudan University Shanghai Cancer Center.

**Results:**

We identified three distinct necroptosis-related classifications (NRCs) with discrepant clinical outcomes and biological functions. Characterization of TME revealed that there were two stable necroptosis-related phenotypes in CRC: a phenotype characterized by few TME cells infiltration but with EMT/TGF-pathways activation, and another phenotype recognized as immune-excluded. NRG_score for predicting survival outcomes was established and its predictive capability was verified. In addition, we found NRCs and NRG_score could be used for patient or drug selection when considering immunotherapy and chemotherapy.

**Conclusions:**

Based on comprehensive analysis, we revealed the potential roles of NRGs in the TME, and their correlations with clinicopathological parameters and patients’ prognosis in CRC. These findings could enhance our understanding of the biological functions of necroptosis, which thus may aid in prognosis prediction, drug selection, and therapeutics development.

**Supplementary Information:**

The online version contains supplementary material available at 10.1186/s12967-022-03431-6.

## Background

Necroptosis is a novel form of regulated necrotic cell death mechanistically mimicking apoptosis and morphologically resembling necrosis [1, 2]. It is mainly regulated by the key proteins such as RIPK1, RIPK3, and their substrate, mixed-lineage kinase domain-like protein (MLKL) [3–5]. Previous researches have reported the relevance of necroptosis in many human diseases, including inflammatory diseases, neurodegenerative diseases, and cancer etc. [6–8]. In addition, it has been suggested to be involved in cancer initiation, progression, immunity, and chemoresistance, providing novel perspectives and potential targets for cancer therapy, for which several therapeutic agents aiming to treat cancer by inducing or manipulating necroptosis are under investigation [6, 9].

Colorectal cancer (CRC) is a major lethal malignancy worldwide [10, 11]. Like other malignancies, tumor microenvironment (TME) plays an indispensable role in CRC tumorigenesis [12]. Previous reports indicated that myeloid-derived suppressive cell (MDSC), an anti-tumor immune suppressor, accumulates in CRC tissue and promotes cancer metastasis [13, 14]. In advanced stage CRC, the well-known immune-activated effectors, CD8^+^ T cells can be suppressed by IL-17A secretion from Th17 cells [15]. As the most exciting breakthrough in cancer treatment, immune-checkpoint blockade (ICB) therapy based on CTLA-4 and PD-1, has demonstrated promising efficacy in CRC patients [16–18]. However, only some of those with microsatellite instability high (MSI-H) or mismatch repair deficient (dMMR) status could benefit from ICB therapy [19]. Therefore, it is necessary and urgent to further investigate the TME characteristics in CRC to identify more effective immunotherapeutic targets.

The involvement of necroptosis has been reported not only in cancer cells but also in other components in the TME [20, 21]. For example, necroptosis could promote pancreatic tumorigenesis by inducing the expression of CXCL1, a potent chemoattractant for myeloid cells that was highly expressed in a RIP1- and RIP3-dependent manner, which could shape the immune suppressing environment [22]. Therefore, further exploring the correlation between TME cells infiltration and necroptosis can provide new perspectives for understanding underlying mechanisms and developing cancer therapeutics, such as combination treatment of necroptosis-based therapy and immunotherapy.

By using bulk and single-cell transcriptomic data analysis, we identified two stable necroptosis-related phenotypes in CRC: a phenotype characterized by few TME cells infiltration but with EMT/TGF-β pathways activation, and another recognized as an immune-excluded phenotype [23]. We further established a scoring system, which could reveal TME characteristics, help accurately determine patients’ survival outcomes, and predict responses to immunotherapy and chemotherapy.

## Materials and methods

### Preparation of bulk RNA expression datasets

A total of 1003 patients from Gene Expression Omnibus (GEO) database (including GSE33113, GSE39582, GSE14333, and GSE37892) were recruited in this study. We corrected the batch effects of GEO datasets using combat method [24] and integrated them into a meta-GEO cohort.

A total 626 patients (578 tumors and 48 normal) in the TCGA cohort were obtained from the UCSC Xena (https://xenabrowser.net/datapages/TCGA-COAD/READ). Somatic mutation data were downloaded from https://portal.gdc.cancer.gov/repository. Copy number variation information was extracted from UCSC Xena. The basic information of these datasets was shown in Additional file 10: Table S1.

### Analysis of single-cell RNA data

Single-cell RNA (scRNA) datasets were downloaded from GEO database (including CRC datasets from GSE144735, GSE178318, LUAD datasets from GSE131907). We calculated the score of single-cell using ‘AddModuleScore’ function via signature α and β.

To calculate the risk score of single-cell data, we first averaged gene expression of each patient to represent their bulk gene expression level. Then we calculated their risk score as follows: risk score = Σ (Expi × coefi), according to methods in necroptosis-related gene score (NRG_score).

### Necroptosis-related genes used for analysis

Thirty-three necroptosis-related genes (NRGs) were retrieved from previous publications [4, 8]. The details of NRGs are shown in Additional file 11: Table S2.

### Consensus molecular clustering by “ConsensusClusterPlus”

We performed consensus clustering with “ConsensusClusterPlus” to identify classifications in CRC patients based on the expression of necroptosis-related genes (NRGs). The final number of clusters was determined by cumulative distribution function (CDF). K = 3 was finally set as the number of clusters. The annotation of clusters of all datasets was shown in Additional file 10: Table S1.

### Gene set variation analysis (GSVA) and single-sample gene set enrichment (ssGSEA) analysis

We calculated pathway activities of tumor samples (Fig. 2E and Additional file 3: Fig. S3C) using GSVA R package. The gene-signatures included for analyzing were downloaded from Hallmark gene sets and C2 curated gene sets (MSigDB database v7.4) [25].

We evaluated immune cell types signature scores using ssGSEA analysis. The immune cell types signature was extracted from the study of Charoentong [26].

### CMS classification for bulk RNA-seq

We utilized CMSclassifier [27] to classify TCGA-COAD/READ tumor samples. The CMS subtypes of TCGA and GEO databases were shown in Additional file 10: Table S1.

### TME infiltration evaluation using ssGSEA, CIBERSORT and ESTIMATE

We adopted the CIBERSORT [28] deconvolution approach to evaluating the relative abundance of 22 tumor-infiltrating immune cells (TIICs). To confirm the stable TME infiltration patterns of necroptosis-related clusters, we also evaluated immune cell infiltration with cell types from the study of Charoentong [26] using ssGESA analysis [29]. In addition, we used ESTIMATE algorithm to calculate tumor purity, immune and stromal scores of each patient.

### Somatic mutation analysis

Varscan file format of somatic mutation data were downloaded from https://portal.gdc.cancer.gov/repository. Copy number variation information was curated from UCSC Xena online. Maftool R package was used to identify mutant genes and calculate TMB level.

### Quantitative real-time polymerase chain reaction (RT-qPCR)

We collected 208 pairs of patients’ tissues (including CRC and adjacent non-tumor tissues) from Fudan University Shanghai Cancer Center (FUSCC) in this study. The written informed consent was signed by all patients according to the Institutional Review Boards of FUSCC, and the study was approved by the Ethical Committee of FUSCC.

RNA was extracted from these samples by using TRIzol reagent (Invitrogen, Carlsbad, CA, USA), which was then reversed into complementary DNA (cDNA) with a PrimeScript RT reagent kit (Takara). Then RT-qPCR was performed using SYBR-Green assays (Takara). The data were calculated using the 2^−ΔΔCt^ value, and normalized with 18 s rRNA. The primer sequences used in our study are shown in Additional file 15: Table S6.

### Construction of the prognostic NRG_score

NRG_score was calculated to quantify the expression patterns of NRGs the individual samples. First, the differentially expressed genes (DEGs) were subjected to univariate Cox regression analysis to identify those linked to CRC overall survival. Second, the patients were classified into different necroptosis phenotype-related groups (gene-cluster A, gene-cluster B, and gene-cluster C) for deeper analysis using an unsupervised clustering method based on the expression of prognostic DEGs (Additional file 13: Table S4) and 33 NRGs. Finally, based on necroptosis phenotype-related prognostic genes, the Lasso Cox regression algorithm was used to minimize the risk of over-fitting using the “glmnet” R package [30]. We analyzed the change trajectory of each independent variable and then used tenfold cross-validation to establish a model. As previously reported [31], we totally performed 1000 iterations and included 5 gene groups for further screening. A gene model with 13 genes showed the highest frequencies of 726 compared to other four-gene models (Fig. 5A). Thus, this 13-gene model was applied to generate the gene signature for calculating NRG_score, which was calculated as follows:

$$\mathrm{NRG}\_\mathrm{score }=\Sigma (\mathrm{Expi }\times \mathrm{ coefi})$$

Based on the median risk score, a total of 578 patients in the training set were divided into low-risk and high-risk groups in survival analysis. Similarly, the testing and all sets were divided into low- and high-risk groups, each of which was subjected to Kaplan–Meier survival analysis and the generation of receiver operating characteristic (ROC) curves. The NRG_score of TCGA and GEO datasets were shown in Additional file 14: Table S5.

### Drug susceptibility analysis

To explore the differences in the therapeutic effects of drugs in CRC patients, we calculated the drug imputed sensitivity score of drugs from Sanger’s Genomics of Drug Sensitivity in Cancer (GDSC) v2 using the “oncoPredict” package [32].

### Kaplan–Meier survival analysis

We plotted the Kaplan–Meier (K-M) survival curve using R package ‘Survminer’ (0.4.6). We stratified samples into high and low gene expression subgroups using surv-cutpoint function.

### Statistical analyses

Statistical analysis was performed using R (version 4.0.0) and GraphPad Prism (version 7.04). The Wilcox test, log rank test, Kruskal–Wallis H test, and Pearson’s Chi-square test were performed in this study. Detailed descriptions of statistical tests are specified in the figure legends.

## Results

### Landscape of genetic variation of NRGs in CRC

**Fig. 1:**
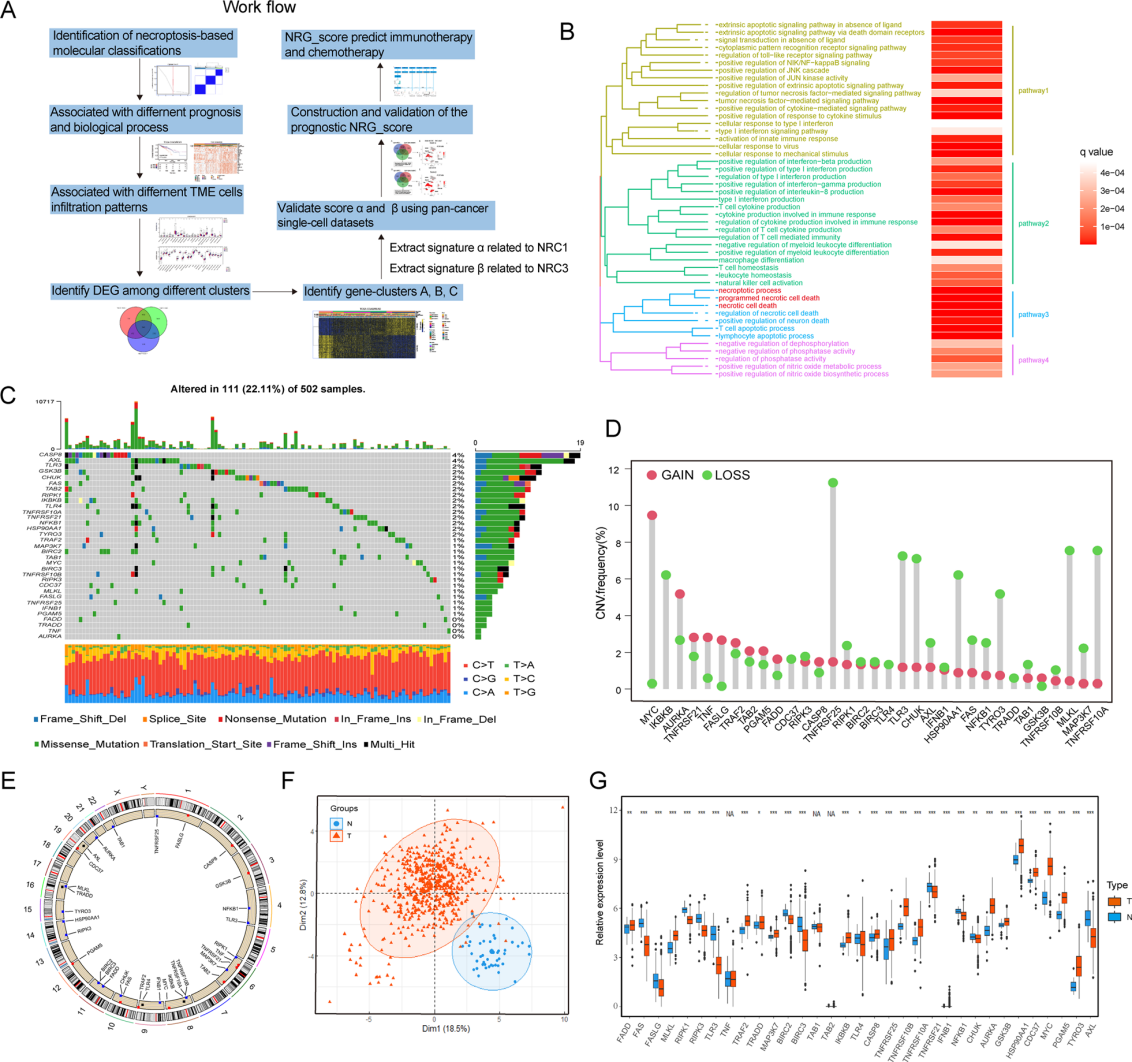
Landscape of genetic variation of necroptosis-related genes in colorectal cancer. **A** A flowchart of our study. **B** Gene ontology annotation of necroptosis-related genes. **C** Oncoplot show genetic alterations of 33 NRGs in CRC. The number on the right indicated the mutation frequency in each gene. Each column represented individual patients. **D** CNV frequency of 33 NRGs in CRC tumors. **E** Locations of CNV alterations in NRGs on 23 chromosomes. **F** Principal component analysis of necroptosis-related genes to distinguish tumors from normal samples in TCGA-COAD/READ cohort. All samples: n = 626; tumor: n = 578; normal: n = 48. **G** Expression distributions of 33 NRGs between normal and CRC tissues

A flowchart of our research was shown in Fig. 1A. In this study, we investigated the roles of 33 NRGs (Additional file 11: Table S2) in CRC. As expected, gene ontology (GO) enrichment analysis showed that these genes were characterized by the biological processes of cell death, especially necroptosis (Fig. 1B). Then, frequency of somatic mutations of NRGs in CRC was analyzed (Fig. 1C). A total of 111 out of 502 CRC samples in TCGA cohort showed genetic alterations of NRGs. Among them, CASP8 had the highest mutation rate (4%), while four NRGs (FADD, TRADD, TNF and AURKA) didn’t present any mutation. Further analysis of copy number variation (CNV) mutation revealed the prevalent copy number alterations in these NRGs (Fig. 1D). The locations of CNV alterations on chromosomes were shown in Fig. 1E. Based on paired tumor-normal sample data, principal component analysis (PCA) was conducted, which showed that NRGs could distinguish CRC samples from normal ones (Fig. 1F). Afterwards, expression of NRGs between CRC and normal samples was compared, revealing that genes with CNV amplification were significantly enriched in tumor samples compared to the normal, such as MYC, FADD, AURKA, TRAF2 and PGAM5, while the expression of TLR3, CHUK, RIPK1, FAS, NFKB1 and AXL was markedly decreased in tumor samples, consistent with that of CNV deletion (Fig. 1G). Taken together, the genetic landscape and expression levels of NRGs between CRC and normal samples were revealed to be significantly different, indicating that necroptosis might play an important role in regulating CRC tumorigenesis.

### Identification of necroptosis-related subtypes in CRC

**Fig. 2:**
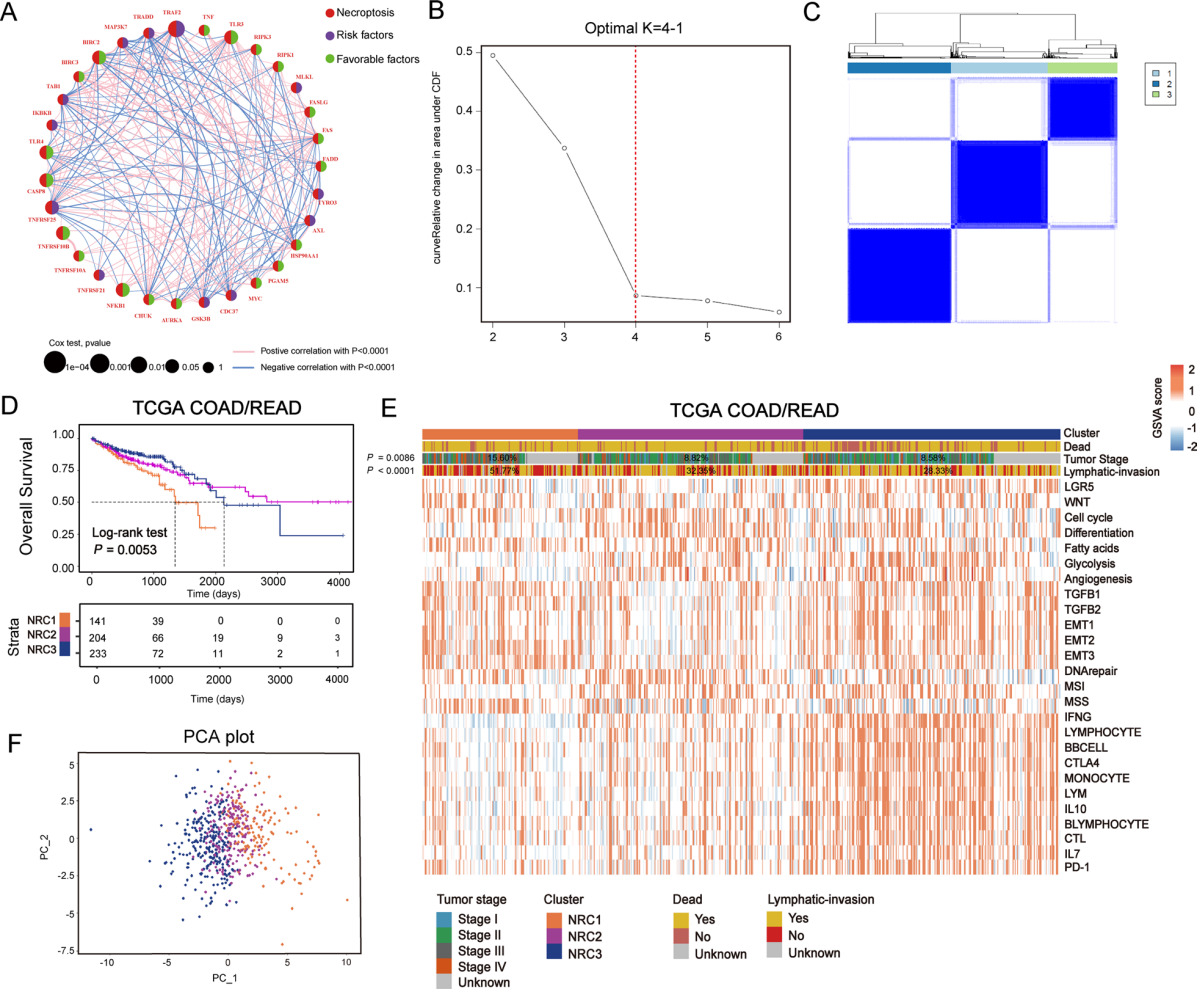
Identification of necroptosis-related subtypes in CRC. **A** Correlation between 33 NRGs. The size of each gene represents survival impact (log-rank test P values indicated). Favorable factors for overall survival indicated in green, and risk factors indicated in purple. The thickness of the line represents the strength of correlation estimated by Pearson correlation analysis. Positive correlation is indicated in pink and negative correlation in blue. **B** Plot shows the cumulative distribution function (CDF) curve. **C** Heatmap shows the consensus matrix heatmap using “ConsensusClusterPlus”. The optimal number of clusters: K = 3. **D** Kaplan–Meier curves for overall survival of three necroptosis-related clusters (NRC) in TCGA. The P value was calculated by the log-rank test. **E** Heatmap shows the differences in clinicopathologic features and expression levels of NRGs between three NRCs. The statistical difference of clinicopathologic features was compared through Pearson’s Chi-square test. **F** Principal component analysis of three NRCs in TCGA-COAD/READ cohort

To comprehensively understand the expression patterns of NRGs involved in tumorigenesis, 1581 patients from five available CRC cohorts (TCGA-COAD/READ, GSE14333, GSE33113, GSE37892 and GSE39582) were integrated in our study for further analyses. The landscape of NRGs interactions, regulator connections, and their prognostic value in CRC patients were demonstrated in a necroptosis network (Fig. 2A). Univariate Cox regression and Kaplan–Meier analysis showed that some of them had prognostic value, and the details were shown in Additional file 10: Fig. S1 and Additional file 12: Table S3. Based on these analyses, seven NRGs (TLR3, TLR4, BIRC2, TRAF2, CASP8, NFKB1 and TNFRSF10B) were identified as prognostic genes.

We next used a consensus clustering algorithm [33] to stratify CRC tumor samples based on the expression of the 33 NRGs (Fig. 2B, C; Additional file 2: Fig. S2A, B). Accordingly, we identified three distinct clusters and referred them as necroptosis-related clusters (NRCs), including 141 cases in NRC1, 204 in NRC2 and 233 in NRC3 (Fig. 2D–F, Additional file 10: Table S1), among which NRC1 and NRC3 had the worse long-term prognosis in TCGA-COAD/READ cohort (Fig. 2D; overall survival (OS), P = 0.0053; log-rank test). In addition, we combined four GEO datasets with available clinical data (GSE33113, GSE39582, GSE14333 and GSE37892) into a meta-GEO cohort and obtained the similar results of classification and prognosis (Additional file 3: Fig. S3B-S3D; relapse-free survival (RFS), P < 0.0001, log-rank test). Moreover, further analysis revealed significantly different distribution of clinicopathological characteristics among different NRCs (Fig. 2E). For example, NRC1 had the most patients with advanced stage disease (stage IV) (15.60%, P = 0.0086, Pearson’s Chi-square test) and lymphatic invasion (51.77%, P < 0.0001, Pearson’s Chi-square test), evidencing why it showed the worst prognosis.

To understand the biological discrepancies among the three distinct clusters, we performed gene set variation analysis (GSVA) [34] on tumor samples (Fig. 2E and Additional file 3: Fig. S3A, C, D). The results showed that NRC1 and NRC3 were enriched in pathways mainly correlated with tumor-specific and stromal pathways such as TGF-β and epithelial-mesenchymal transition (EMT), supporting their poor prognosis. Interestingly, among the three clusters, NRC3 was remarkably enriched with immune cells and immunotherapy-related pathways, such as lymphocyte, monocyte, PD-1 and CTLA4 signaling. All of these findings indicated the marked differences in the intrinsic biological underpinnings of the three NRCs in CRC.

### Distinct tumor microenvironment infiltration in NRCs

**Fig. 3:**
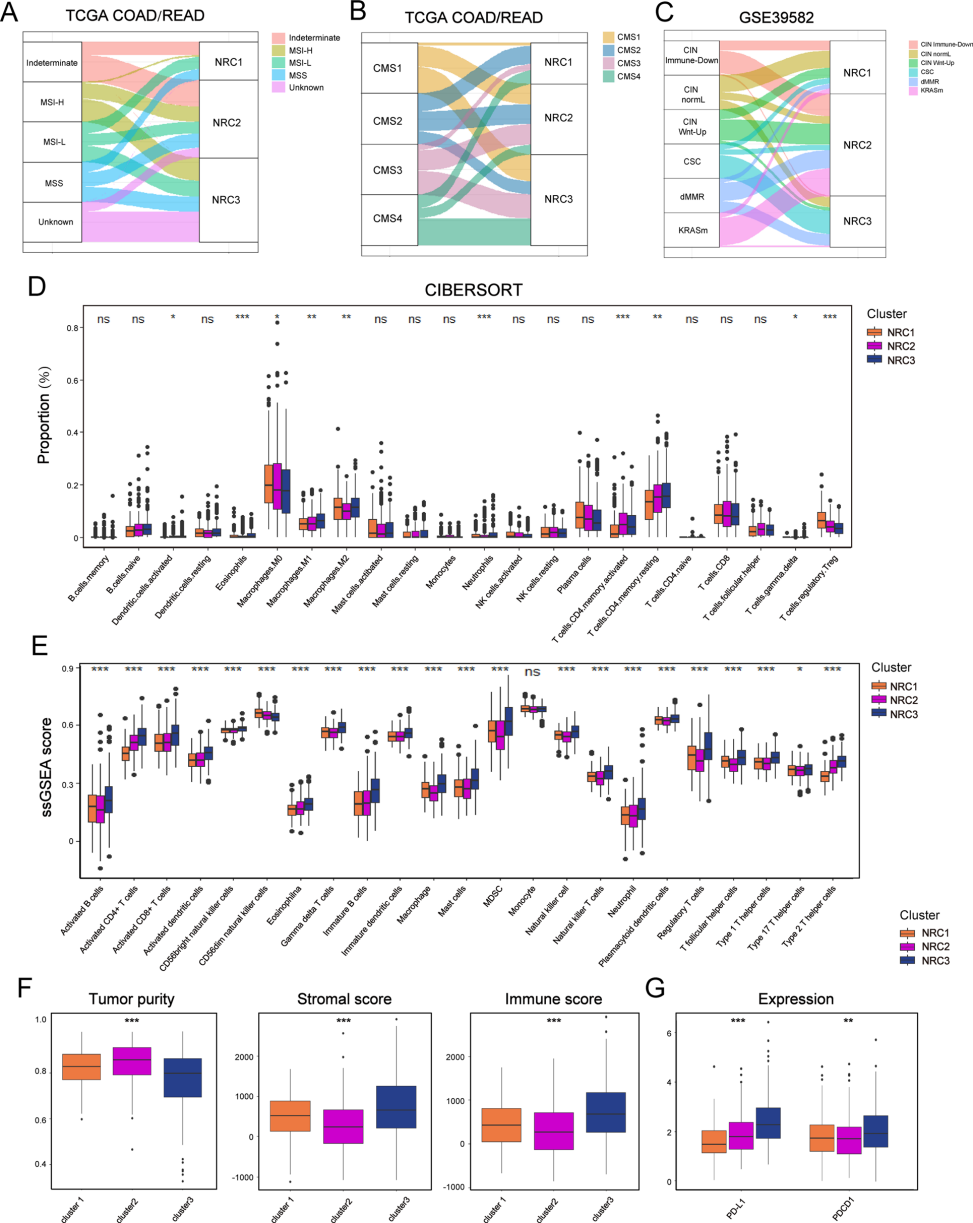
Distinct tumor microenvironment infiltration in necroptosis-related clusters. **A–C** Alluvial diagram of clusters in groups with different molecular subtypes. **D** Relative abundance of 22 tumor-infiltrating immune cells (TIICs) of three clusters in TCGA cohort. The statistical difference of three clusters was compared through the Kruskal–Wallis H test. *P < 0.05; **P < 0.01; ***P < 0.001. **E** Barplot shows the ssGSEA score of immune cell subtypes from the study of Charoentong in three necroptosis-related clusters. The statistical difference of three clusters was compared through the Kruskal–Wallis H test. *P < 0.05; **P < 0.01; ***P < 0.001. **F** Tumor purity, immune and stromal score of three NRCs in TCGA cohort. The statistical difference of three clusters was compared through the Kruskal–Wallis H test. *P < 0.05; **P < 0.01; ***P < 0.001. **G** Comparison of PD-L1 and PDCD1 (PD-1) expression between three NRCs. The difference of three clusters was compared through the Wilcox test. *P < 0.05; **P < 0.01; ***P < 0.001

Previous studies have indicated MSI-H/ dMMR status could predict the response to immunotherapy in CRC [16]. We next explored the MSI/MMR status in tumor samples of NRCs, which showed that MSI-H was mainly concentrated within NRC2 and NRC3 (Fig. 3A). When the association of NRCs with the consensus molecular subtype (CMS) system was analyzed, it revealed that CMS1-immune subtype was mainly clustered into NRC2 and NRC3 (Fig. 3B). In GSE39582 cohort, samples with dMMR status were predominantly grouped into NRC2 and NRC3 (Fig. 3C). Notably, CMS4 and CSC subtypes, characterized by prominent transforming growth factor-β (TGF-β) activation, stromal invasion and angiogenesis [26], were mainly concentrated within NRC3 (Fig. 3C).

To further characterize the microenvironment heterogeneity of NRCs, we performed CIBERSORT [28] and ssGSEA analyses (Fig. 3D, E; Additional file 4: Fig. S4A). The results showed that not only antitumor immune cell populations such as memory CD4^+^ T cells and activated CD4^+^/CD8^+^ T cells, but also immune-suppressive cells such as MDSC and regulatory T cells were enriched within NRC3. Moreover, we used the ESTIMATE algorithm [35] to quantify the overall infiltration of immune cells (Immune score), stromal cells (Stromal score) and tumor cell purity (Tumor purity) across three NRCs (Fig. 3F and Additional file 4: Fig. S4B). Here we demonstrated that NRC3 encompassed low tumor purity, and displayed remarkable stromal cells infiltration. Taken together, NRC3 was considered as an immune-excluded phenotype characterized by stromal activation and weakened immune cell infiltration. However, there was no significant difference in immune cell infiltration between NRC1 and NRC2 by CIBERSORT and ssGSEA analyses. Using ESTIMATE algorithm, we observed that NRC2 had higher tumor purity than NRC1, while NRC1 displayed stronger stromal cells infiltration than NRC2 (Fig. 3F and Additional file 4: Fig. S4B). These features were not consistent with MSI-H/CMS1-like characteristic of NRC2, which were shown in Fig. 3A–C. As previously reported, the expression of PD-1/PD-L1 could predict the response to immunotherapy in some cancers [36]. We next compared the PD-1/PD-L1 expression level among the three NRCs and observed the highest expression in NRC3 (Fig. 3G and Additional file 4: Fig. S4C). However, considering the immune-excluded phenotype of NRC3, patients in NRC3 might display ineffective response to anti- PD-1/PD- L1 treatment, which might partially explain why high expression of PD-1/PD-L1 has not been clinically demonstrated to effectively predict immunotherapy response in CRC.

### Necroptosis phenotype-related DEGs in CRC

**Fig. 4:**
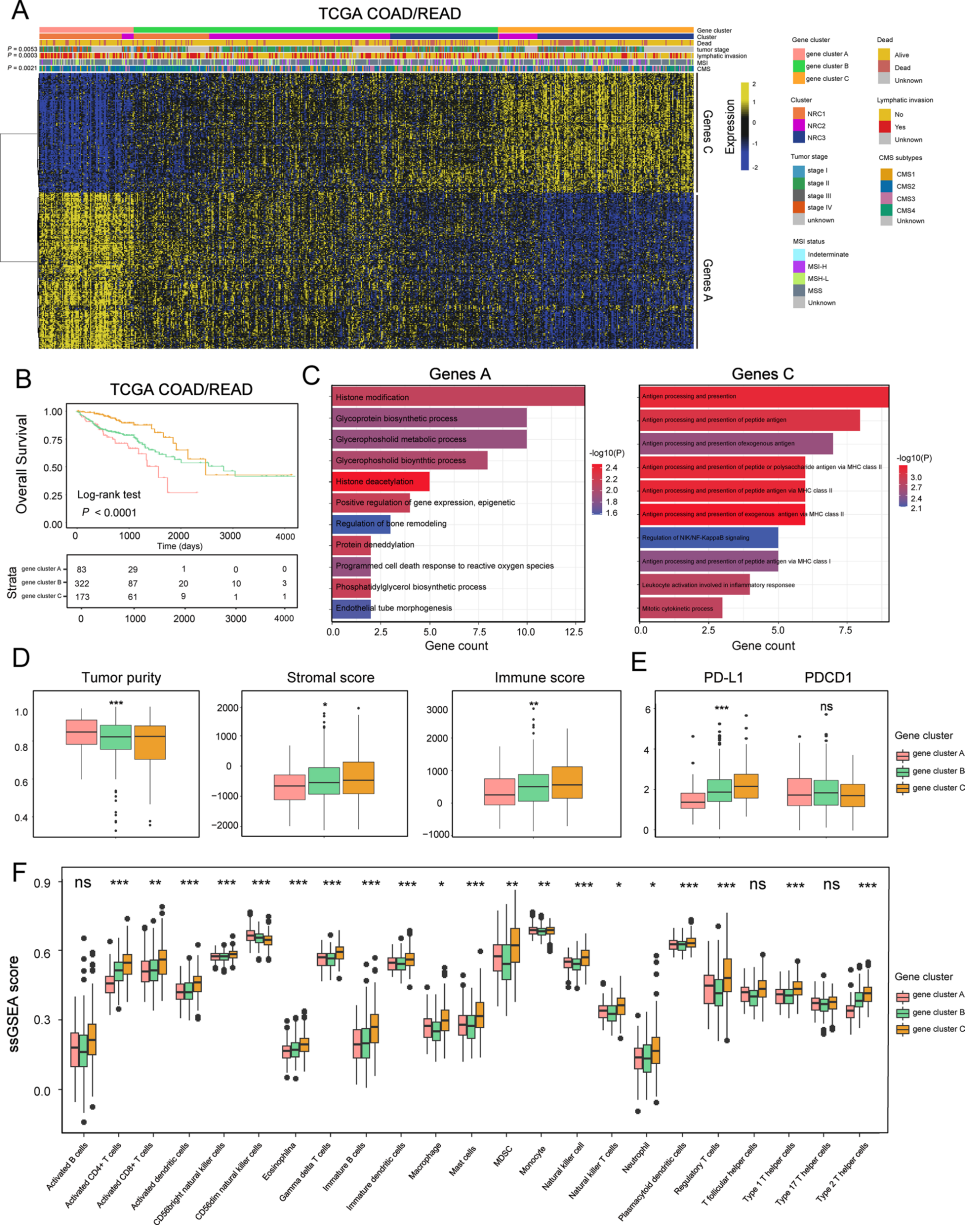
Necroptosis phenotype-related DEGs in colorectal cancer. **A** Consensus clustering of TCGA tumor samples using necroptosis phenotype-related signature. Clinical and molecular characteristics are shown on the top. The difference of three gene clusters was compared through the Pearson’s Chi-square test. **B** Kaplan–Meier curves for overall survival of three gene clusters in TCGA. The P value was calculated by the log-rank test. **C** Gene ontology enrichment (GO) analysis of genes A and genes C. **D** Tumor purity, immune and stromal score of three gene clusters in TCGA cohort. The statistical difference of three clusters was compared through the Kruskal–Wallis H test. *P < 0.05; **P < 0.01; ***P < 0.001. **E** Comparison of PD-L1 and PDCD1 (PD-1) expression between three gene clusters. The difference of three clusters was compared through the Wilcox test. *P < 0.05; **P < 0.01; ***P < 0.001. **F** Barplot shows the ssGSEA score of immune cell subtypes from the study of Charoentong in three gene clusters. The statistical difference of three clusters was compared through the Kruskal–Wallis H test. *P < 0.05; **P < 0.01; ***P < 0.001

To further confirm the underlying molecular and clinical patterns determined by NRGs, we overlapped 2862 DEGs among the three NRCs and recognized them as necroptosis phenotype-related signature (Additional file 5: Fig. S5). We next included these DEGs for univariate Cox regression analysis and obtained 475 prognostic genes. Then, we performed unsupervised consensus clustering analysis based on these 475 prognostic genes and divided TCGA patients into three necroptosis phenotype-related signature groups with different clinicopathologic subgroups, which were defined as gene-cluster A, B and C (Fig. 4A; Additional file 10: Table S1). By hierarchical clustering and gene ontology enrichment (GO) analysis (Fig. 4C), 475 prognostic genes were only grouped into signature genes A and C (Additional file 13: Table S4). Genes A were clustered into gene-cluster A and associated with metabolic processes and stromal biological processes such as endothelial tube morphogenesis. Genes C were enriched within gene-cluster C and associated with immune cells activation and antigen processing. We observed that gene-cluster A presented the worst prognosis (Fig. 4B; overall survival (OS), P < 0.0001, log-rank test) with the highest proportion of advanced stage patients (stage IV) (15.66%, P = 0.0053, Pearson’s Chi-square test) (Fig. 4A) and the most patients with lymphatic invasion (51.81%, P = 0.0003, Pearson’s Chi-square test). We also found that gene-cluster A contained the most NRC1 tumors, while gene-cluster C had most of the NRC3 tumors (Fig. 4A). For CMS subtypes (Fig. 4A), CMS4 was mainly grouped into gene-cluster C, consistent with the pattern of NRC3 (28.32% in gene-cluster C, P = 0.0021, Pearson’s Chi-square test). Subsequent ESTIMATE analysis showed that gene-cluster C had low tumor purity and remarkable stromal cells infiltration (Fig. 4D). Moreover, gene-cluster C displayed the highest expression level of PD-1/PD-L1, similar to NRC3 (Fig. 4E). For TME cell infiltration (Fig. 4F), both adaptive and innate immune cells were enriched in gene-cluster C. Overall, based on necroptosis-related genes, there were two stable distinct phenotypes in CRC: like NRC1, gene-cluster A was characterized by few TME cells infiltration (Figs. 3F and 4D) but with EMT/ TGF-β pathways activation, and like NRC3, gene-cluster C was characterized by remarkable stromal, immune cells infiltration, and EMT/TGF-β activation, which was similar to CMS4-like and thus recognized as an immune-excluded phenotype[23].

### Single-cell analysis of NRCs

**Fig. 5:**
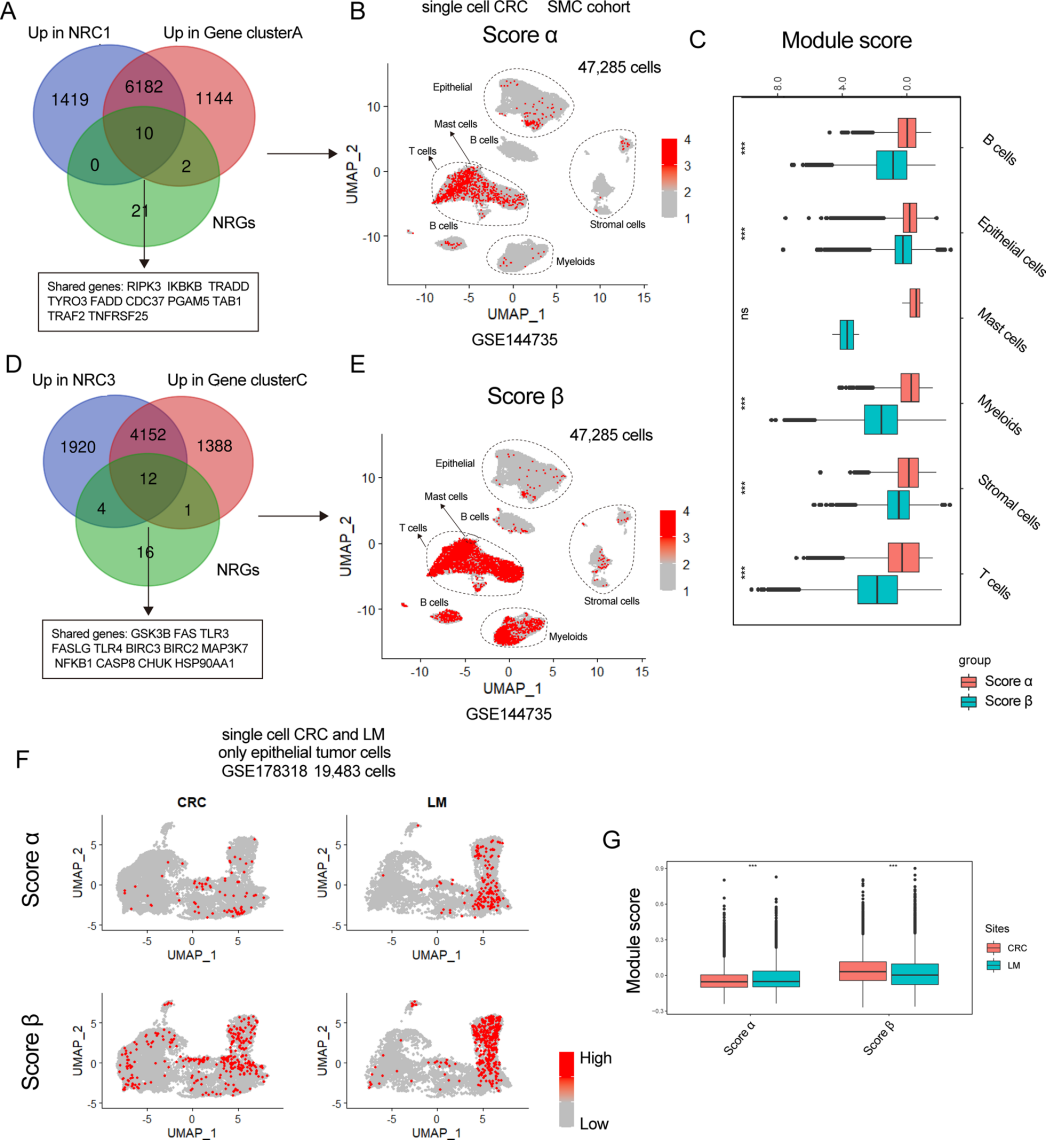
Single-cell analysis of necroptosis-based classification in CRC. **A** Venn plot shows overlapped genes between NRC1, gene-cluster A and NRGs. **B** UMAP plot show score α in 47,285 single cells of SMC cohort. **C** Box-plot shows score of two signatures in different cell types of SMC cohort. The statistical difference of two groups was compared through the Wilcox test. *P < 0.05; **P < 0.01; ***P < 0.001. **D** Venn plot shows overlapped genes between NRC3, gene-cluster D and NRGs. **E** UMAP plot shows score β in 47,285 single cells of SMC cohort. **F** UMAP plot shows score α and β in 19,796 epithelial cells of GSE178318. **G** Box-plot shows score of two signatures in different sites of GSE178318. The statistical difference of two groups was compared through the Wilcox test. *P < 0.05; **P < 0.01; ***P < 0.001

To further understand biological and TME characteristics of NRC1 and NRC3, we analyzed single-cell datasets of CRC (GSE144735 [37] and GSE178318 [38]). We first overlapped representative genes of NRC1, gene-cluster A and NRGs, and obtained a total of 10 genes (RIPK3, IKBKB, TRADD, TYRO3, FADD, CDC37, PGAM5, TAB1, TRAF2, TNFRSF25; Fig. 5A) which were recognized as signature α. Identical method was performed on NRC3, and 12 genes were identified as signature β (GSK3B, FAS, TLR3, FASLG, TLR4, BIRC3, BIRC2, MAP3K7, NFKB1, CASP8, CHUK, HSP90AA1; Fig. 5D). We then used these two signatures to score single-cell data of SMC and KUL CRC cohorts from GSE144735 (Fig. 5B, E, Additional file 6: Fig S6A and S6B). The results showed that score β in TME cells (especially in stromal and T cells) were higher than score α (Fig. 5C and Additional file 6: Fig. S6C). Therefore, NRC3 and gene-cluster C were indeed infiltrated by stromal and immune cells, consistent with an immune-excluded phenotype. Just as previously reported [37, 39], the strong stromal cell infiltration pattern might cause the CMS4-like phenotype and EMT/TGF-β activation in NRC3 and gene-cluster C.

Next, we included a single-cell dataset (GSE178318) which contained liver metastasis and scored a total of 19,483 tumor epithelial cells using signature α and β (Fig. 5F). We found that score α in epithelial cells from liver metastasis was higher than that from CRC primary sites, while score β in liver metastasis was lower than primary sites (Fig. 5G), indicating that high score α might predict high risk of CRC liver metastasis. Because EMT is a crucial step that promotes tumor metastasis [40], we postulated that EMT phenotype of NRC1 was mainly exhibited on tumor cells, while EMT phenotype of NRC3 was caused by its stromal cell infiltration.

**Fig. 6:**
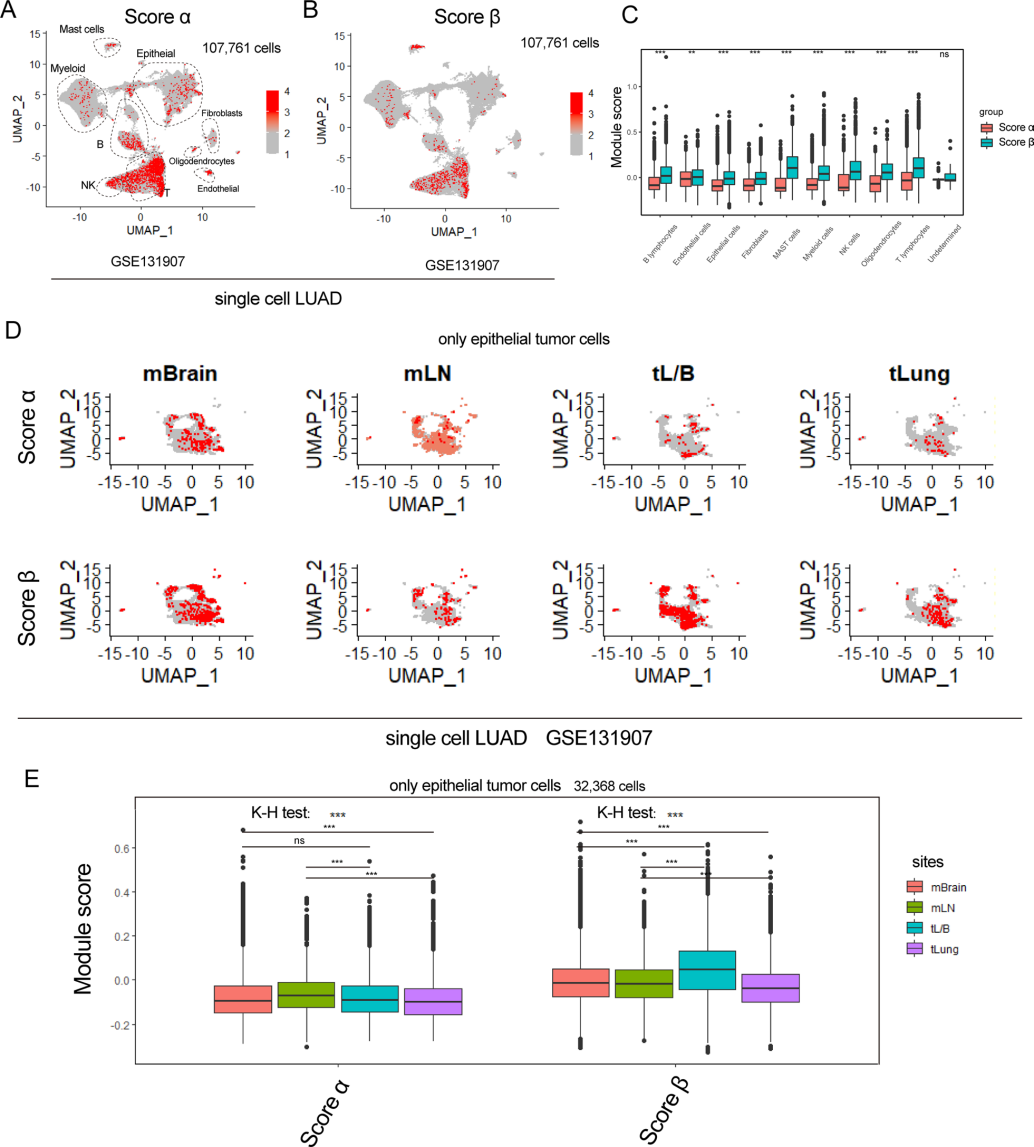
Single-cell analysis of necroptosis-based classification in LUAD. **A**, **B** UMAP plot show score α and β in 107,761 single cells of GSE131907. **C** Box-plot shows score of two signatures in different cell types of GSE131907 cohort. The statistical difference of two groups was compared through the Wilcox test. *P < 0.05; **P < 0.01; ***P < 0.001. **D** UMAP plot shows score α and β in epithelial cells of GSE131907. **E** Box-plot shows score of two signatures in different sites of GSE131907

Finally, we would like to explore whether these interesting findings could be replicated in other cancer. We performed identical analyses on single-cell data of metastatic lung adenocarcinoma (LUAD) (GSE131907) [41]. We found that score β were higher in TME cells (Fig. 6A–C). Then, we extracted tumor epithelial cells from early-(tLung), advanced-stages(tL/B), metastatic lymph nodes (mLN) and brain metastases (mBrain). By scoring tumor cells using the two signatures (Fig. 6D), we observed that score α in mBrain was significantly higher than primary site tLung (Fig. 6E). Score α in mLN was significantly higher than primary sites including tLung and tL/B (Fig. 6E). However, score β was the highest in advanced-stage primary sites (tL/B; Fig. 6E). All these results were similar to that in CRC datasets. Taken together, there were indeed two stable patterns based on necroptosis-related genes.

### Construction and validation of the prognostic NRG_score

**Fig. 7:**
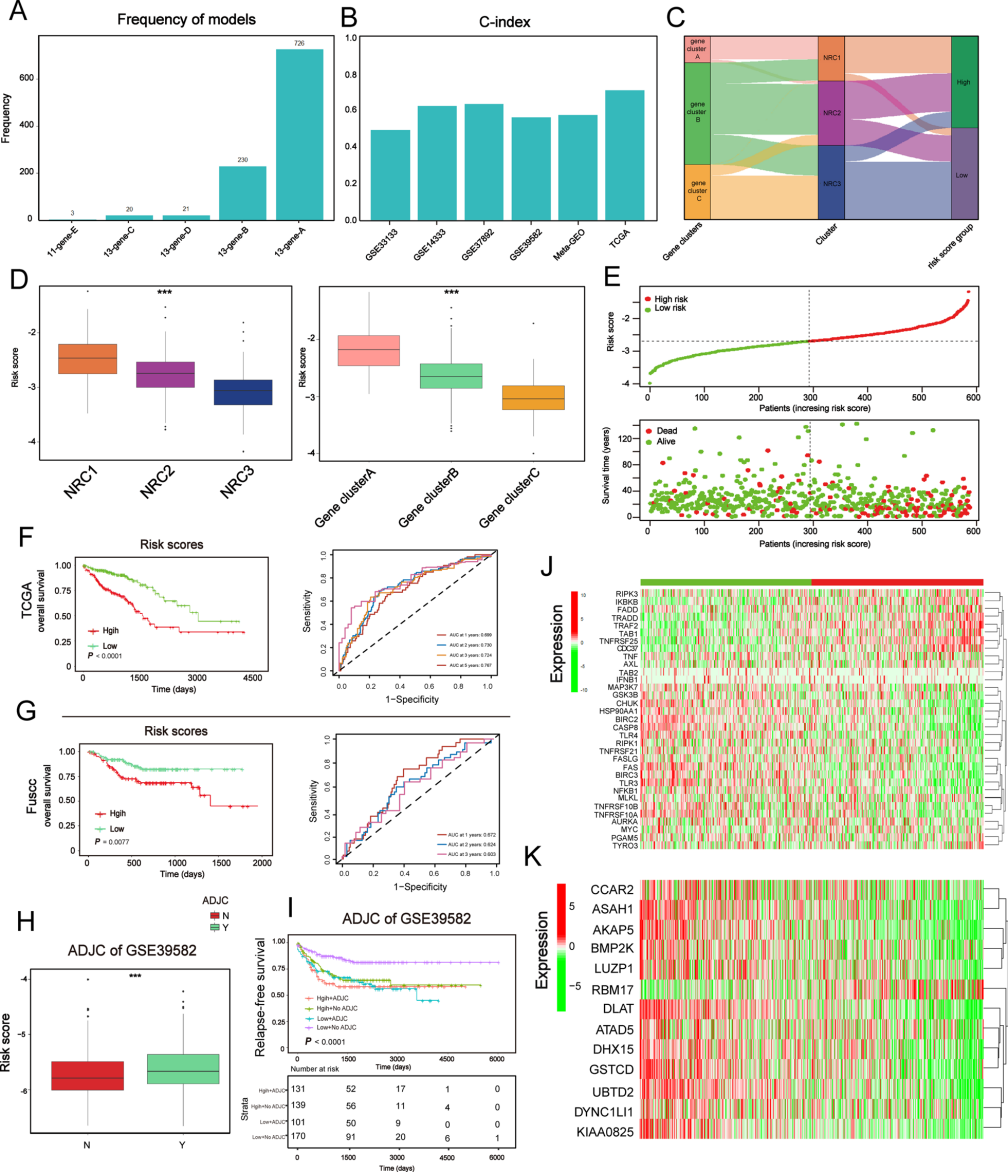
Construction and validation of the prognostic NRG_score. **A** Generation of the ten gene groups after 1000 iteration. The gene model with 13 genes was selected to construct the signature for NRG_score as its highest frequencies of 726 compared to other four gene models. **B** The c‑index of both training and testing sets. **C** Alluvial diagram of NRCs in groups with different gene clusters and NRG_score groups. **D** Barplots show the risk score between three NRCs and three gene clusters. The statistical difference of three clusters was compared through the Kruskal–Wallis H test. *P < 0.05; **P < 0.01; ***P < 0.001. **E** Ranked dot and scatter plots showing the NRG_score distribution and patient survival status. **F, G** Kaplan–Meier analysis of the survival rate between the two groups. The high and low groups were divided by the median value of the NRG_score (left pannael). ROC curves to predict the sensitivity and specificity of 1-, 2-, 3-, and 5-year survival according to the NRG_score (right panel). **H** Barplot shows the NRG_score between groups with adjuvant chemotherapy (ADJC) and without adjuvant chemotherapy (ADJC). The statistical difference of two clusters was compared through the Wilcox test. *P < 0.05; **P < 0.01; ***P < 0.001. **I** Survival analysis among four patient groups stratified by both NRG_score and treatment with adjuvant chemotherapy (ADJC). **J**, **K** Differences in the expression of 33 NRGs and 13 genes among the two gene subtypes. The statistical difference of two groups was compared through the Wilcox test. *P < 0.05; **P < 0.01; ***P < 0.001

A flowchart illustrating the generation of the signature for NRG_score was presented in Additional file 7: Fig. S7A-B. As previously reported [30, 31], we conducted 1000 iterations in total and 5 gene groups were included for further screening. A gene model with 13 genes showed the highest frequencies of 726 compared to other four gene models (Fig. 7A), for which it was further applied to generate the gene signature for NRG_score calculation. We then calculated the c-index to validate the accuracy of NRG_score in survival prediction. The c-index for TCGA dataset, meta-GEO, GSE33113, GSE14333, GSE37892 and GSE39582 were 0.702, 0.568, 0.468, 0.621, 0.630, and 0.555, respectively (P < 0.05, Fig. 7B). The high-risk group in TCGA dataset, meta-GEO, GSE14333, GSE37892 and GSE39582 had worse survival rate than the low-risk group (Additional file 7: Fig. S7B). These results demonstrated the predictive power of the signature for survival in 5 datasets except in GSE33113. Finally, we constructed the NRG_score as follows:

$$\begin{aligned} {\text{Risk score}} & = \left( { - 0.00{\text{4956222}} \times {\text{DHX15 expression}}} \right) + \left( { - 0.{\text{115492238}} \times {\text{ BMP2K expression}}} \right) \\ & + \left( { - 0.0{\text{43519529}} \times {\text{ LUZP1 expression}}} \right){\text{ }} + {\text{ }}\left( { - 0.0{\text{74852629}} \times {\text{ GSTCD expression}}} \right) \\ & + \left( { - 0.{\text{3737}}0{\text{8136}} \times {\text{DLAT expression}}} \right) + \left( {0.{\text{68}}0{\text{1}}0{\text{8}}0{\text{72}} \times {\text{ RBM17 expression}}} \right) \\ & + \left( { - 0.0{\text{66367539}} \times {\text{ATAD5 expression}}} \right) + \left( { - 0.{\text{159671175}} \times {\text{AKAP5 expression}}} \right) \\ & + \left( { - 0.0{\text{57824168}} \times {\text{DYNC1LI1 expression}}} \right) + \left( { - 0.{\text{19987373}}0 \times {\text{KIAA}}0{\text{825 expression}}} \right) \\ & + \left( { - 0.{\text{3}}00{\text{275421}} \times {\text{UBTD2 expression}}} \right) + \left( { - 0.{\text{2}}0{\text{865}}0{\text{641}} \times {\text{CCAR2 expression}}} \right) \\ & + \left( { - 0.0{\text{33744746}} \times {\text{ASAH1 expression}}} \right). \\ \end{aligned}$$

We next explored the differences in NRG_score between NRCs, and between necroptosis phenotype-related gene clusters, which showed the highest NRG_score in NRC1 and gene-cluster A, consistent with results of prognosis (Fig. 7C, D). The distribution plot of the risk of NRG_score showed that death rate increased with the increase of NRG_score (Fig. 7E). The survival analysis revealed that patients with low NRG_score showed improved overall survival (log-rank test, P < 0.0001; Fig. 7F). Additionally, the 1-, 2-, 3-, and 5-year survival rates of NRG_score were reflected by AUC values of 0.699, 0.730, 0.724, and 0.767, respectively (Fig. 7F). Subsequently, we validated the prognostic predictive ability of the NRG_score in external datasets (Meta-GEO, GSE14333, GSE37892), which showed that patients could be dichotomized into low- and high-risk subgroups by using the aforementioned formula of the training set (Additional file 8: Fig. S8A-S8B). Moderate AUC values were reproduced in GSE14333 and GSE37892 when it comes to the prediction of the 1-, 2-, 3-, and 5- year survival using the NRG_score (Additional file 8: Fig. S8C). In addition, we also plotted K-M survival curves and calculated the AUC values of a cohort from FUSCC based on NRG_score. The results showed that high-risk score group displayed a worse prognosis (log-rank test, P = 0.0077; Fig. 7G) and AUC values at 1-, 2-, 3-year were 0.672, 0.624 and 0.603, respectively. Taken together, the NRG_score could be applied to predict the survival of CRC patients.

**Fig. 8:**
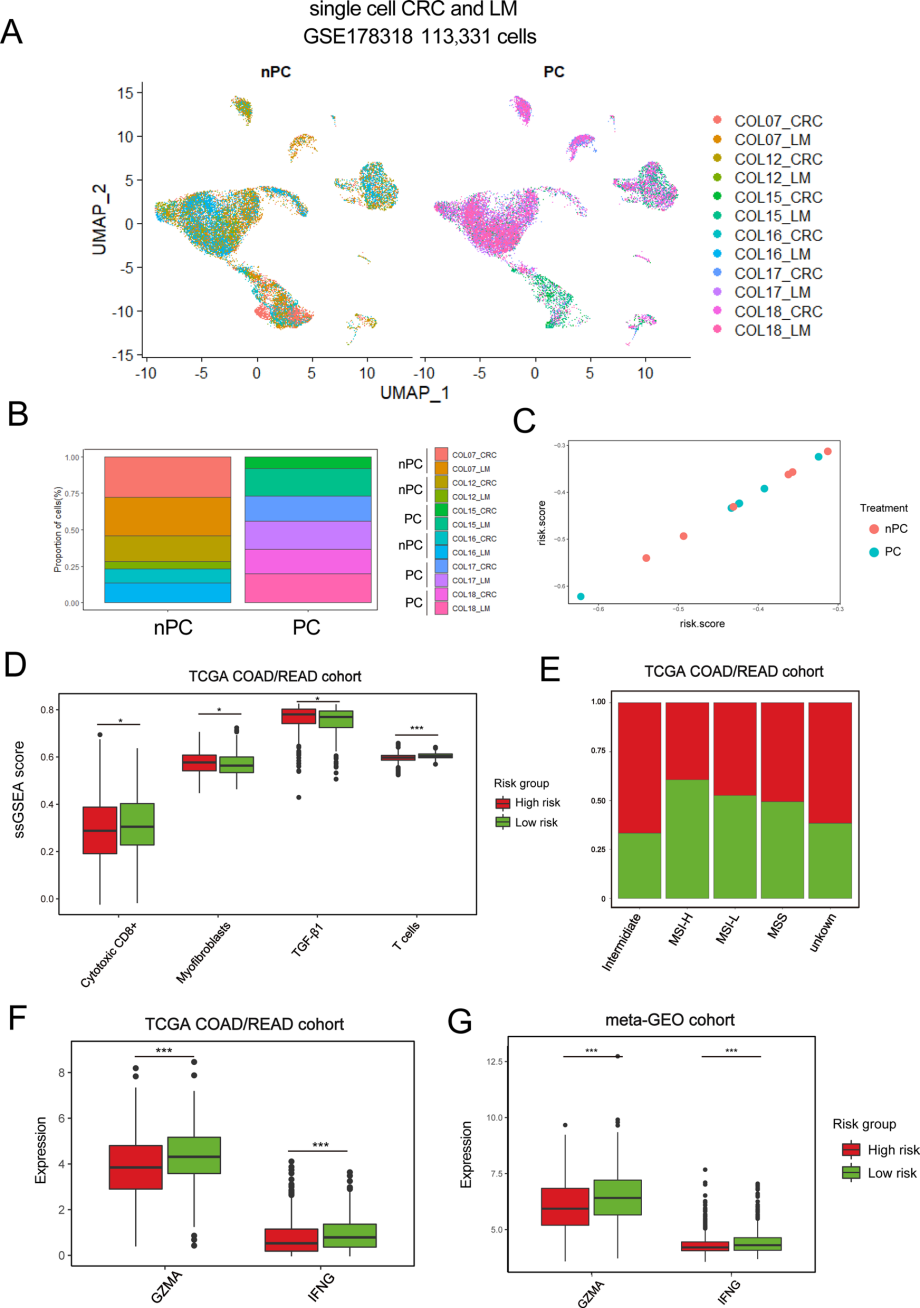
Evaluation of TME between the high- and low-risk groups. **A** UMAP plot shows 113,331 single cells of GSE178318 cohort. **B** Bar-plot shows the proportion of samples corresponding to treatment (PC: Preoperative chemotherapy; nPC: non-Preoperative chemotherapy). **C** Dot plot shows the distribution of samples from GSE178318 based on their risk score. **D** Score of immune-related gene-signatures between high- and low-risk groups. **E** Differences of molecular subtypes between low- and high-risk groups. **F, G** Expression of GZMA and IFNG between low- and high-risk groups. The statistical difference of two clusters was compared through the Wilcox test. *P < 0.05; **P < 0.01; ***P < 0.001

Because GSE39582 contained the patients who underwent adjuvant chemotherapy, we then examined whether the NRG_score could predict the response to adjuvant chemotherapy (ADJC). The results showed that patients receiving chemotherapy had the higher NRG_score (Fig. 7H). Subsequent survival analysis showed that low score group without ADJC manifested better overall survival. However, patients receiving ADJC in both high and low score group had poor survival (Fig. 7I). As presented above, patients with high NRG_score coupled with more advanced stage disease, which might partially explain why patients with high score and receiving ADJC showed poor survival. However, the result of patients with low score and receiving ADJC might indicate that these patients might not benefit from ADJC. We also calculated risk score (see methods) in single-cell dataset curated from GSE178318 [38], which contained three patients treated with chemotherapy (PC: Preoperative chemotherapy) (COL15, COL17, and COL18) and three patients were treatment naïve (COL07, COL12, and COL16) (Fig. 8A, B). We observed that most of the treated samples’ scores were high, which was similar to bulk transcriptomic analysis (Fig. 8C).

Finally, we assessed the transcriptional signature between high and low NRG_score groups. The expression levels of 33 NRGs and 13 model genes between high- and low-risk groups in TCGA and meta-GEO cohort were shown in Fig. 7J, K and Additional file 8: Fig. S8D, E.

### Evaluation of TME between the high- and low-risk groups

As presented by the immune scores of representative gene-signatures in Fig. 8D, high NRG_score was negatively related to T cells and cytotoxic CD8^+^ T cells, while it was positively correlated with myofibroblasts and TGF-β pathway, suggesting high-score group exhibited a suppressive immune microenvironment. For molecular classifications, we observed that low NRG_score group was enriched with more MSI-H tumors (Fig. 8E). Since infiltration level of cytotoxic CD8^+^ T cells predicted the response to immunotherapy, we explored the relationship between NRG_score and representative genes of cytotoxic CD8^+^ T cells, such as GZMA and IFNG (Fig. 8F, G). The results showed that low NRG_score group showed upregulation of GZMA and IFNG (Fig. 8F, G). These results suggested patients in low-score group might exhibit effective response to immunotherapy because of its high infiltration level of cytotoxic CD8^+^ T cells and MSI-H status.

### Imputed drug sensitivity score in necroptosis-related phenotypes

**Fig. 9:**
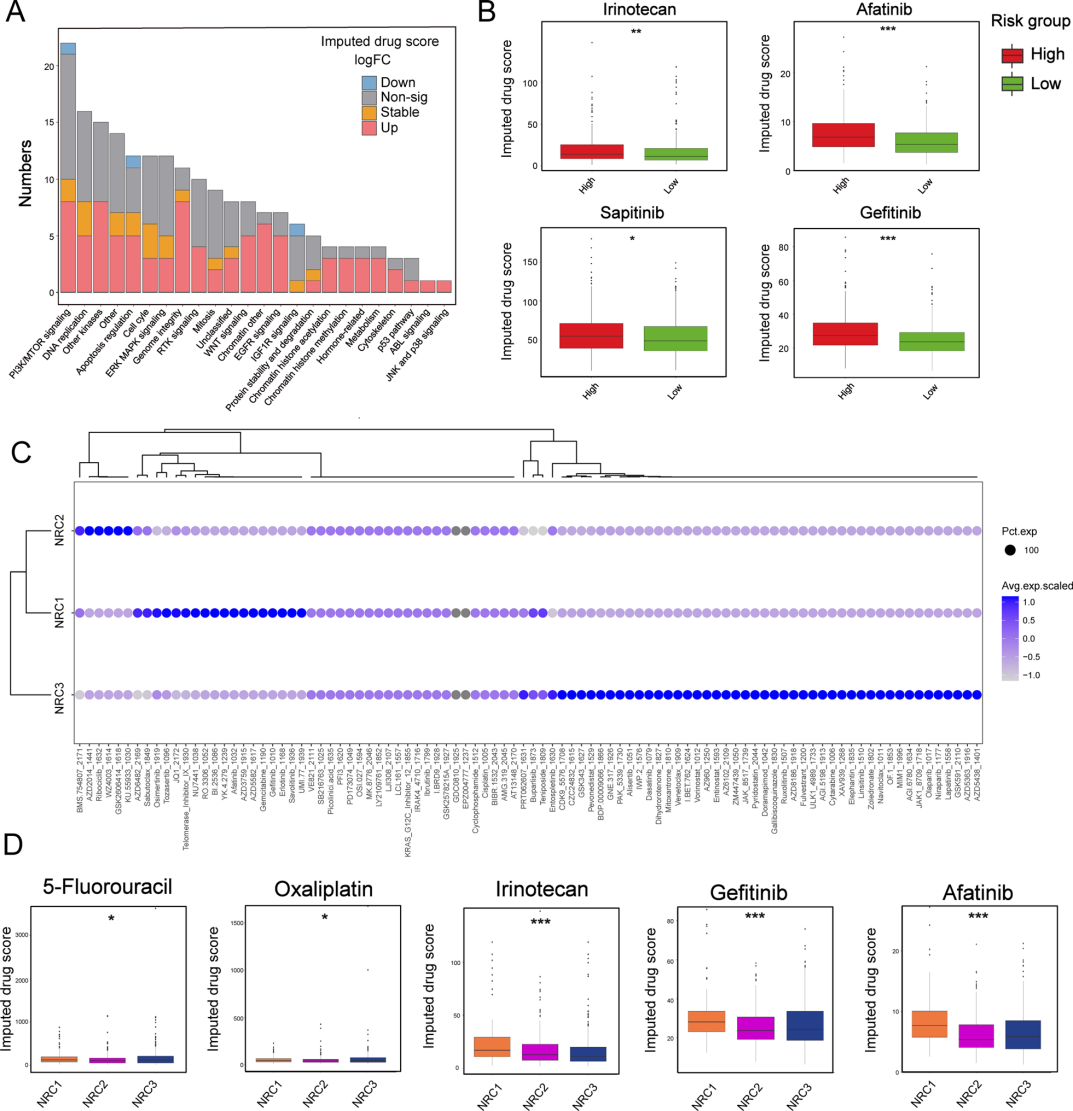
Imputed drug sensitivity score of necroptosis-related phenotype. **A** The number of drugs in GDSC v2 that was significantly upregulated or downregulated (P < 0.05) in the high-risk score group versus low-risk score group among each of 24 drug categories in the TGCA cohort. **B** Barplot shows the imputed drug sensitivity score between high- and low-risk groups. The statistical difference of two clusters was compared through the Wilcox test. *P < 0.05; **P < 0.01; ***P < 0.001. **C** Dot plot shows the imputed drug sensitivity score among three NRCs. **D** Barplot shows the imputed drug sensitivity score among three NRCs. The statistical difference of three clusters was compared through the Kruskal–Wallis H test. *P < 0.05; **P < 0.01; ***P < 0.001

We next evaluated the differences in drug susceptibility between the high-and low-risk groups. Differential analysis demonstrated that the imputed scores of 89 drugs from Sanger’s Genomics of Drug Sensitivity in Cancer (GDSC) v2 [42] were significantly different (with imputed score elevation of 86 drugs and decline of 3 drugs) in CRC tumors in reference to normal samples (Fig. 9A). Afterwards, we selected drugs currently adopted to treat CRC in clinical practice to evaluate the drug sensitivity of patients in the high- and low-risk groups [32] (Fig. 9B). Interestingly, we found that patients in the high-risk group had higher imputed score for irinotecan, afatinib, sapitinib and gefitinib, suggesting that these patients might not respond to the aforementioned drugs effectively (Fig. 9A, B). Thus, patients with different NRG_score might respond to drugs differently.

We also evaluated the drug susceptibility among the three NRCs. The imputed score of 190 drugs were shown in Fig. 9C. Our results showed that there were significant differences in imputed score of 5-Fluorouracil, Oxaliplatin, Irinotecan, Gefitinib and Afatinib among the three NRCs (Fig. 9D). For example, high imputed score of 5-Fluorouracil and Oxaliplatin in NRC3 suggested that these patients might not respond effectively to these two drugs, while high score indicated that patients in NRC1 might not respond to Irinotecan, Gefitinib, and Afatinib (Fig. 9D). Taken together, these results indicated that patients within different NRCs might present discrepant sensitivity to chemotherapeutic drugs.

### Developing a nomogram to predict patients’ survival

**Fig. 10:**
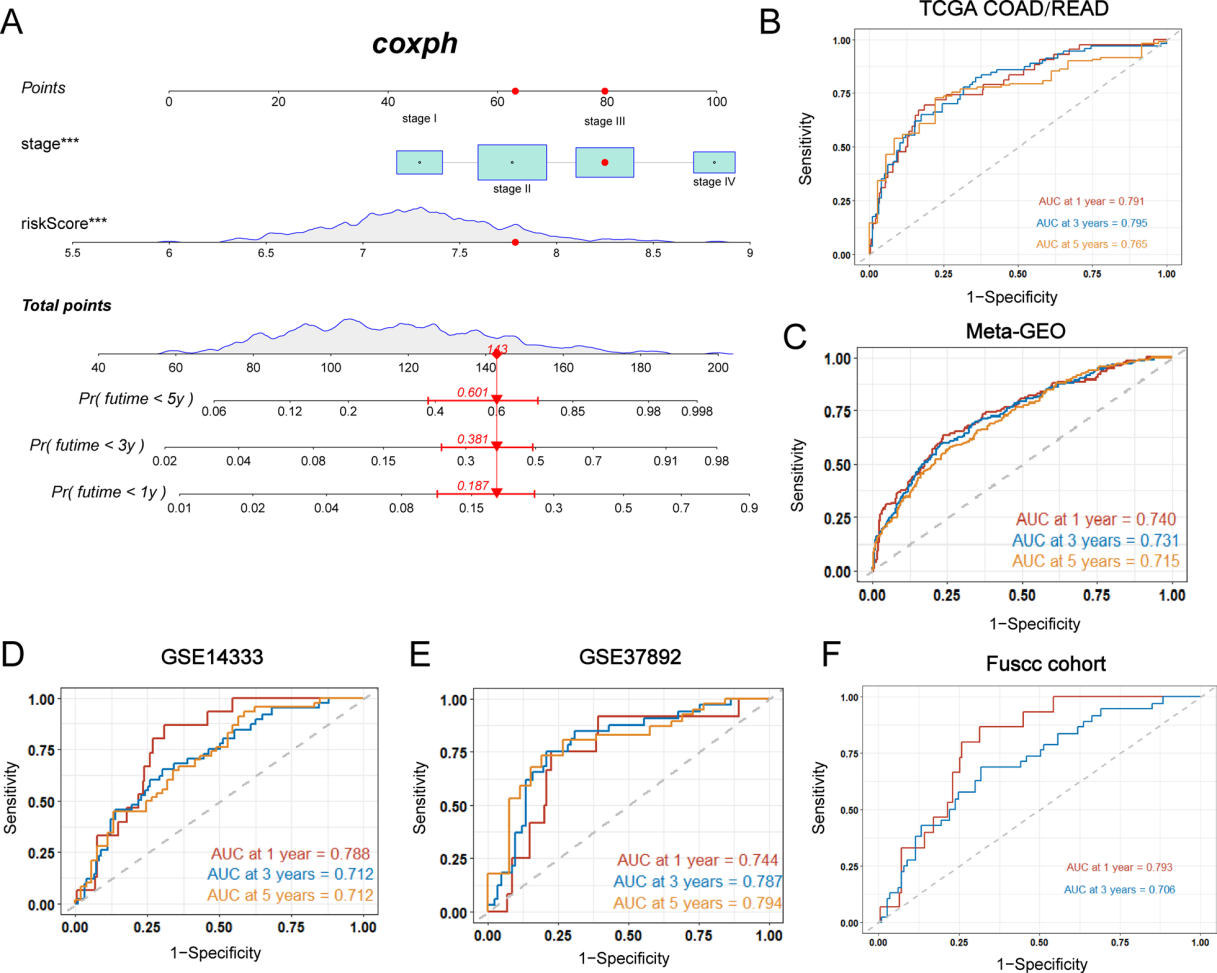
Developing a nomogram to predict patients’ survival. **A** Nomogram for predicting the 1-, 3-, 5-, and 10-year RFS of CRC patients in the training set. **B–F** ROC curves for predicting the 1-, 3- and 5-years, ROC curves in the training (TCGA), testing (meta-GEO), GSE37892, GSE14333 and FUSCC cohorts

Next, NRG_score and disease stage (TNM stage) were incorporated to establish a nomogram to predict the 1-, 3-, and 5-year RFS, the results of which were shown in Fig. 10A. The AUC of the nomogram model for survival at 1, 3, and 5 years showed high accuracy in the training set (TCGA), testing set (meta-GEO), and three validation sets (GSE14333, GSE37892 and FUSCC cohorts) (Fig. 10B–F). The predictive accuracy of the nomogram showed AUC values at 1-, 3-, and 5-year in TCGA were 0.791, 0.795 and 0.765, respectively. In the testing set (meta-GEO), the 1-, 3- and 5-year AUC values of the nomogram were 0.740, 0.731, and 0.715, respectively. AUC values of TCGA at 1- and 3- year in this part were higher than that based on only NRG_score in Fig. 7F. AUC values of nomogram (at 1-, 3- or 5-year) in three validation sets (GSE14333, GSE37892 and FUSCC cohorts) were also higher than that based on only NRG_score in Additional file 8: Fig. S8C and Fig. 7G. Furthermore, AUC values of the nomogram at 1-, 3- and 5-year in TCGA, meta-GEO, and GSE14333 sets, and AUC values of the nomogram at 3- and 5-year in GSE37892 were higher than AUC values of TNM stage systems, suggesting that our nomogram displayed an advantage in survival predictive ability over TNM stage systems (Additional file 9: Fig. S9A–D). Subsequently, the calibration plots demonstrated that the nomogram we established could perform similarly in both the training and testing sets (Additional file 9: Fig. S9E–I).

## Discussion

Cell death has recently attracted increasing attention for its potential role in triggering anti-tumor immunity [43]. Like apoptotic cells, emerging researches have showed that necroptotic tumor cells can induce anti-tumor immunity by their interaction with diverse immune cell types [44, 45]. Although various studies have revealed the regulation of NRGs in TME [46, 47], a landscape of TME characteristics mediated by NRGs have not been comprehensively understood.

In this study, we introduced necroptosis-related phenotypes of TME in CRC. Based on 33 NRGs and DEGs associated with necroptosis-related phenotypes, we could stratify CRC samples into three molecular phenotypes (NRC1-3). However, we observed that only two classifications kept stable according to their immune infiltration patterns. Therefore, we postulated that there were two stable TME patterns mediated by necroptosis in CRC: a phenotype characterized by few TME cells infiltration but with EMT/TGF-β pathways activation, and another phenotype characterized by remarkable stromal cells infiltration, together with EMT, TGF-β signaling pathway activation, corresponding to the immune-excluded and CMS4-like phenotype. To confirm these two stable phenotypes related to necroptosis, we performed single-cell transcriptomic analyses in CRC datasets and further validated in LUAD datasets. We observed that score of NRC1 represented by score α was increased in tumor metastatic sites, while score β was elevated in TME cells. We thus postulated that EMT phenotype in NRC1 was mainly exhibited on tumor cells, while CMS4-like and EMT phenotype in NRC3 were predominantly caused by its remarkable stromal cell infiltration. What’s more, high α score might be used to predict the risk of CRC metastasis.

Previous reports suggested that immune context of TME could promote EMT. MDSCs, well-known as immature immune cells, are associated with poor prognosis of cancers for suppressing T cells activation [48]. TGF-β production from MDSCs have been experimentally proved to render a profound impact on tumor metastasis [49]. Stromal cells such as fibroblasts have been also reported as a major source of TGF-β production [50, 51]. TGF-β expressed by cancer-associated fibroblast (CAF) (such as myofibroblast) induces recruitment of more fibroblasts, and might thus lead to a pro-tumorigenic and immunotolerant status [52]. Adaptive immune cells like CD8^+^ T cells respond to TGF-β may also cause an immunosuppressive environment. Since NRC3 was infiltrated by stromal cells and MDSCs, patients in NRC3 cannot respond to PD-1/PD-L1 therapy. Fortunately, NRC3 was remarkably infiltrated by activated T cell populations such as CD4^+^ and CD8^+^ T, which should have been related to anti-tumor immunity. High expression of PD-1/PD-L1 was observed in NRC3, which has been reported to predict response to immune checkpoint inhibitors [53]. Therefore, intervention targeting on stromal cells and MDSCs, and downregulation of TGF-β may help patients within NRC3 regain an effective response to immunotherapy. Without considering TME, the role of necroptosis in tumor cells has not been comprehensively understood either [54]. Previous findings showed that RIPK3 was upregulated in late-stage breast tumors, implying a promising role of necroptosis in tumor progression [54, 55]. In NRC1, we observed upregulation of RIPK3 (Fig. 5A), EMT activity (Additional file 3: Fig. S3A and S3D), and enrichment of advanced stages (15.60%; Fig. 2E), suggesting that RIPK3 may play an indispensable role in CRC progression. Emerging evidences demonstrated that RIPK3 upregulation could potentiate chemotherapeutic effects by inducing necroptosis [56]. Therefore, RIPK3 may be a key mediator resulting in EMT and chemo-sensitive phenotype of patients within NRC1. Future experimental researches are required to investigate the key regulator RIPK3 in CRC development.

We also constructed a robust and effective prognostic NRG_score and demonstrated its predictive ability in CRC survival by integrated analyses of public databases and a patients’ cohort from FUSCC. Patients with low- and high-risk NRG_score displayed significantly different clinicopathological characteristics, prognosis, immune infiltration and drug susceptibility. We observed that high-risk score group was highly infiltrated by myofibroblast and characterized by TGF-β pathway activation. In contrast, low-risk group was enriched with more cytotoxic T cells. We further explored cytotoxic genes like GZMA and IFNG in public database, confirming the precise predictive ability of low-risk score in response to immunotherapy. Interestingly, the exploration of drug imputed score showed patients in high- and low-risk groups might present different chemotherapeutic efficacy, suggesting that NRG_score could be used for patient selection when considering ADJC and there might be potential molecular targets based on NRGs. Finally, by integrating NRG_score and tumor stage, we established a quantitative nomogram, which further improved the performance and facilitated the use of NRG_score. Overall, the NRG_score we constructed can be an accurate prognostic model for prognosis stratification of CRC patients, and a good predictor for immunotherapy and chemotherapy.

In a nutshell, we comprehensively analyzed the mutations and expression patterns of NRGs in CRC. NRCs and NRG_score were established and their associations with TME were explored. Sensitivity to chemotherapy and response to immunotherapy were probed. These integrated analyses highlighted the main role of necroptosis in TME infiltration of CRC. Moreover, we put forward specific genes related to EMT phenotype on tumor cells, and genes related to stromal cells infiltration in TME, which will provide an interesting insight into the mechanism between necroptosis and TME infiltration. However, there are still some limitations: (1) the study was conducted based on retrospective data, thus, selection bias might be unavoidable; (2) though we validated our findings in validation sets based on public datasets, validation in prospective study will further add credibility to these findings; (3) molecular mechanisms of these observations necessitate exploration in the future.

## Supplementary Information

**Additional file 1.** Figure S1. Kaplan-Meier curves of 33 NRGs in TCGA cohort. The surv_function of the R package survminer was used to determine the optimal cutoff value to divide samples into high and low groups.

**Additional file 2.** Figure S2. Identification of necroptosis-related subtypes in CRC, related to Figure 1 (**A**) Heatmap representation of consensus clustering for necroptosis-related genes in TCGA cohort with cluster numbers from 2 to 6. (**B**) Heatmap representation of consensus clustering for necroptosis-related genes in meta-GEO cohort with cluster numbers from 2 to 6.

**Additional file 3.** Figure S3. Clinical characteristics and biological molecular changes underlying three clusters in CRC, related to Figure 2 (**A**) Barplot shows the GSVA score of pathways in three NRCs of TCGA cohort. The statistical difference of three clusters was compared through the Kruskal-Wallis H test. *P < 0.05; **P < 0.01; ***P < 0.001. (**B**) Kaplan-Meier curves for overall survival of three necroptosis-related clusters (NRC) in meta-GEO. The P value was calculated by the log-rank test. (**C**) Heatmap shows the differences in clinicopathologic features and expression levels of NRGs between three NRCs in meta-GEO cohort. (**D**) Barplot shows the GSVA score of pathways in three NRCs of meta-GEO cohort. The statistical difference of three clusters was compared through the Kruskal-Wallis H test. *P < 0.05; **P < 0.01; ***P < 0.001.

**Additional file 4.** Figure S4. Distinct tumor microenvironment infiltration in necroptosis-related clusters, related to Figure 3 (**A**) Barplot shows the ssGSEA score of immune cell subtypes from the study of Charoentong in three necroptosis-related clusters. The statistical difference of three clusters was compared through the Kruskal-Wallis H test. *P < 0.05; **P < 0.01; ***P < 0.001. (**B**) Tumor purity, immune and stromal score of three NRCs in TCGA cohort. The statistical difference of three clusters was compared through the Kruskal-Wallis H test. *P < 0.05; **P < 0.01; ***P < 0.001. (**C**) Comparison of PD-L1 expression between three NRCs. The difference of three clusters was compared through the wilcox test. *P < 0.05; **P < 0.01; ***P < 0.001.

**Additional file 5.** Figure S5. DEGs among the three necroptosis-related clusters

**Additional file 6.** Figure S6. Single-cell analysis of necroptosis-based classification in CRC, related to Figure 5 (**A**-**B**) UMAP plot show score α and β in 17,678 single cells of KUL cohort. (**C**) Box-plot shows score of two signatures in different cell types of KUL cohort. The statistical difference of two groups was compared through the wilcox test. *P < 0.05; **P < 0.01; ***P < 0.001.

**Additional file 7.** Figure S7. Construction and validation of the prognostic NRG_score (**A**-**B**) The workflow of construction and validation of the signature for calculating NRG risk score (NRG_score).

**Additional file 8.** Figure S8. Construction and validation of the prognostic NRG_score, related to Figure 7 (**A**) Kaplan–Meier analysis of the survival rate between the two groups in meta-GEO cohort. (**B**) Ranked dot and scatter plots showing the NRG_score distribution and patient survival status. (**C**) ROC curves to predict the sensitivity and specificity of 1-, 2-, 3-, and 5-year survival according to the NRG_score in GSE14333 and GSE37892 cohort. (**D**-**E**) Differences in the expression of 33 NRGs and 13 genes among the twogene subtypes.

**Additional file 9.** Figure S9. Developing a nomogram to predict patients’ survival, related to Figure 10 (**A**-**D**) ROC curves for predicting the 1-, 3- and 5-years, ROC curves in the training (TCGA), testing (meta-GEO), GSE37892, GSE14333 and FUSCC cohorts based on TNM stage systems (**E**-**I**) Calibration curves of the nomogram for predicting of 1-, 3-, and 5-year survival rate in the training, testing, GSE37892, and GSE14333 sets, and FUSCC cohort.

**Additional file 10.** Supplementary Table S1. The description of patients with bulk RNA expression data, including clinical characteristics and consensus clusters.

**Additional file 11.** Supplementary Table S2. 33 necroptosis-related genes.

**Additional file 12.** Supplementary Table S3. Prognostic values of Necroptosis-related genes

**Additional file 13.** Supplementary Table S4. 475 prognostic genes grouped into signature genes A and C

**Additional file 14.** Supplementary Table S5. NRG_score of TCGA and meta_GEO cohort.

**Additional file 15.** Supplementary Table S6. Primers in this study.

## Data Availability

All data in this study can be obtained from the Gene-Expression Omnibus (GEO; https://www.ncbi.nlm.nih.gov/geo/), the GDC portal (https://portal.gdc.cancer.gov/) and the UCSC Xena (https://xenabrowser.net/datapages/).

## Abbreviations

CRC: Colorectal cancer
LUAD: Lung adenocarcinoma
TMB: Tumor mutation burden
CNV: Copy number variation
GEO: Gene-expression omnibus
GSVA: Gene set variation analysis
HR: Hazard ratios
NRC: Necroptosis-related subtypes
NRG: Necroptosis-related gene
MSI: Microsatellite instability
dMMR: Deficient mismatch repair
TCGA: The Cancer Genome Atlas
TGF-β: Transforming growth factor beta
TME: Tumor microenvironment
