# Supplementary figures and images for: Bulk and single-cell transcriptome profiling reveal necroptosis-based molecular classification, tumor microenvironment infiltration characterization, and prognosis prediction in colorectal cancer
Source: J Transl Med. 2022 May 19;20:235. doi: 10.1186/s12967-022-03431-6 (PMC9118791; doi:10.1186/s12967-022-03431-6)

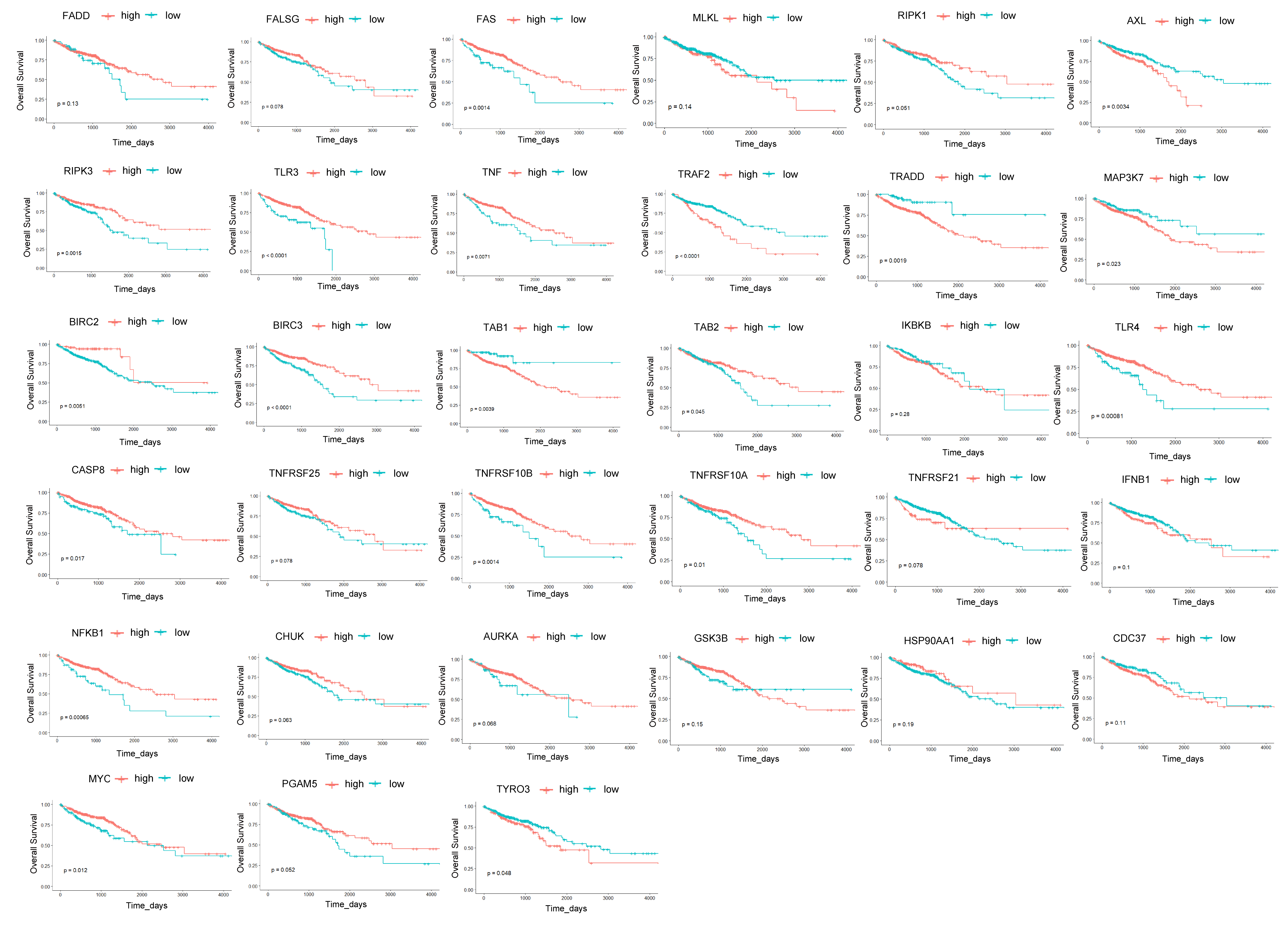

Supplement: Supplementary file 1 — Additional file 1. Figure S1. Kaplan-Meier curves of 33 NRGs in TCGA cohort. The surv_function of the R package survminer was used to determine the optimal cutoff value to divide samples into high and low groups. [file 12967_2022_3431_MOESM1_ESM.tif]

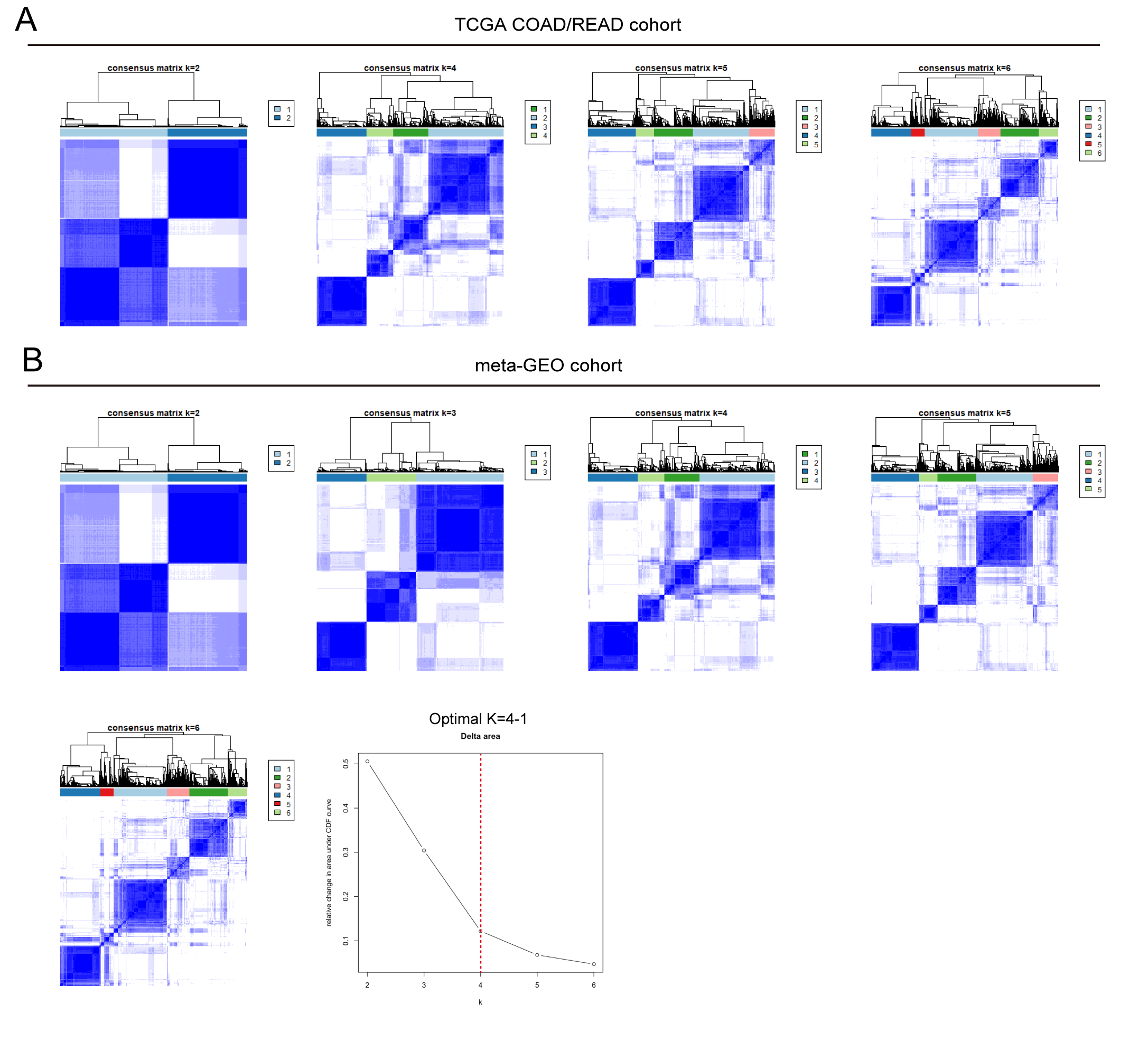

Supplement: Supplementary file 2 — Additional file 2. Figure S2. Identification of necroptosis-related subtypes in CRC, related to Figure 1 (A) Heatmap representation of consensus clustering for necroptosis-related genes in TCGA cohort with cluster numbers from 2 to 6. (B) Heatmap representation of consensus clustering for necroptosis-related genes in meta-GEO cohort with cluster numbers from 2 to 6. [file 12967_2022_3431_MOESM2_ESM.tif]

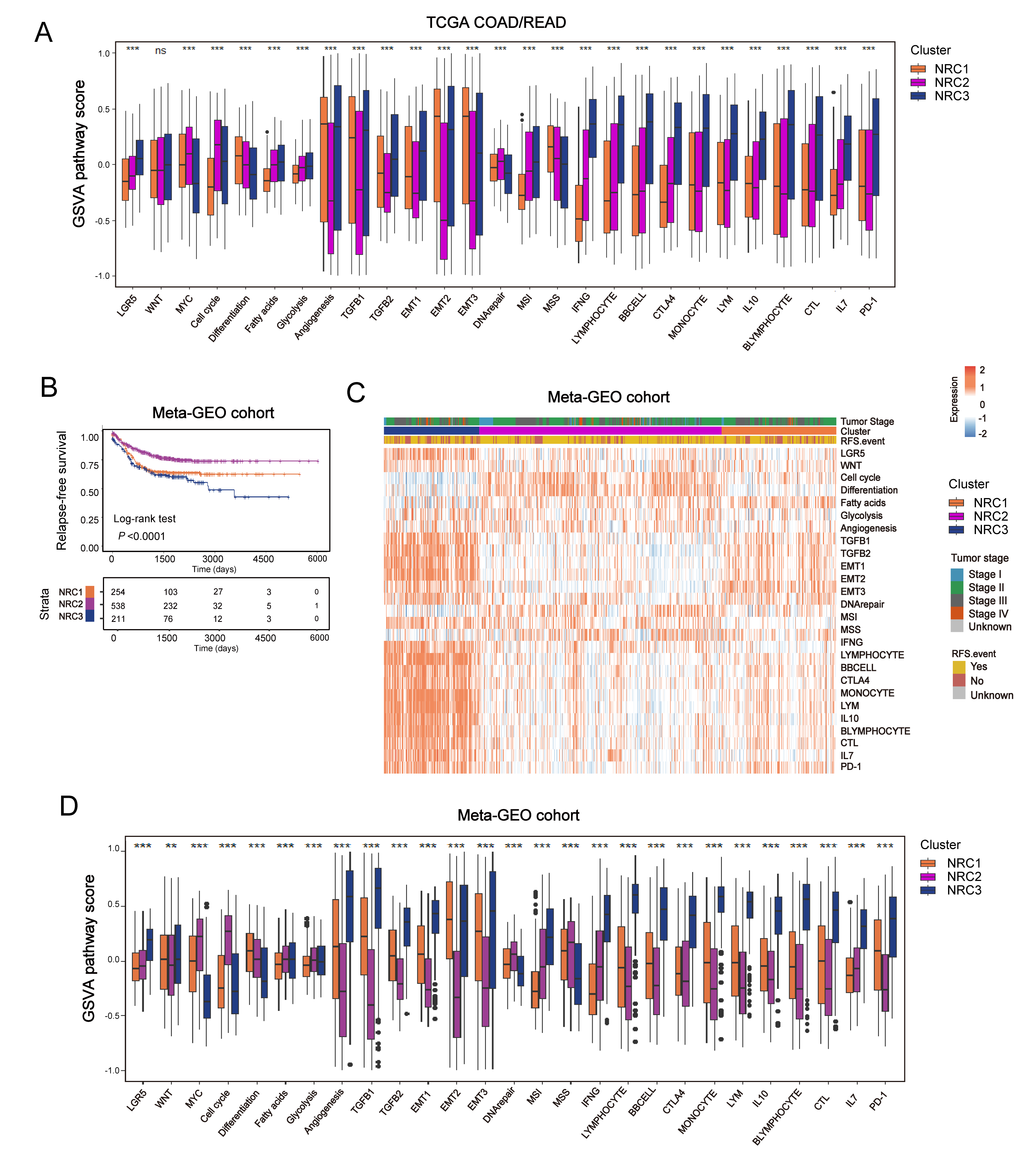

Supplement: Supplementary file 3 — Additional file 3. Figure S3. Clinical characteristics and biological molecular changes underlying three clusters in CRC, related to Figure 2 (A) Barplot shows the GSVA score of pathways in three NRCs of TCGA cohort. The statistical difference of three clusters was compared through the Kruskal-Wallis H test. *P < 0.05; **P < 0.01; ***P < 0.001. (B) Kaplan-Meier curves for overall survival of three necroptosis-related clusters (NRC) in meta-GEO. The P value was calculated by the log-rank test. (C) Heatmap shows the differences in clinicopathologic features and expression levels of NRGs between three NRCs in meta-GEO cohort. (D) Barplot shows the GSVA score of pathways in three NRCs of meta-GEO cohort. The statistical difference of three clusters was compared through the Kruskal-Wallis H test. *P < 0.05; **P < 0.01; ***P < 0.001. [file 12967_2022_3431_MOESM3_ESM.tif]

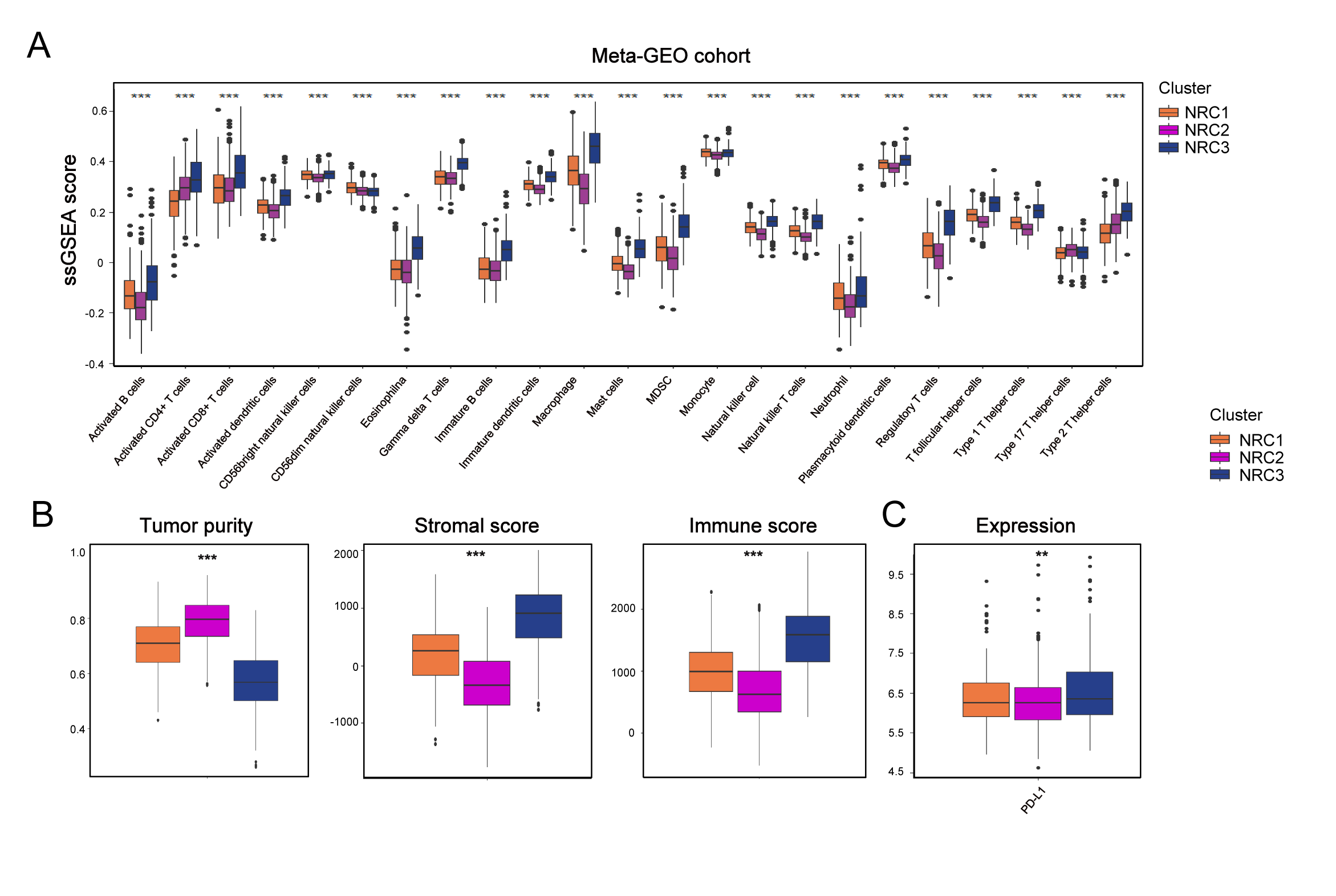

Supplement: Supplementary file 4 — Additional file 4. Figure S4. Distinct tumor microenvironment infiltration in necroptosis-related clusters, related to Figure 3 (A) Barplot shows the ssGSEA score of immune cell subtypes from the study of Charoentong in three necroptosis-related clusters. The statistical difference of three clusters was compared through the Kruskal-Wallis H test. *P < 0.05; **P < 0.01; ***P < 0.001. (B) Tumor purity, immune and stromal score of three NRCs in TCGA cohort. The statistical difference of three clusters was compared through the Kruskal-Wallis H test. *P < 0.05; **P < 0.01; ***P < 0.001. (C) Comparison of PD-L1 expression between three NRCs. The difference of three clusters was compared through the wilcox test. *P < 0.05; **P < 0.01; ***P < 0.001. [file 12967_2022_3431_MOESM4_ESM.tif]

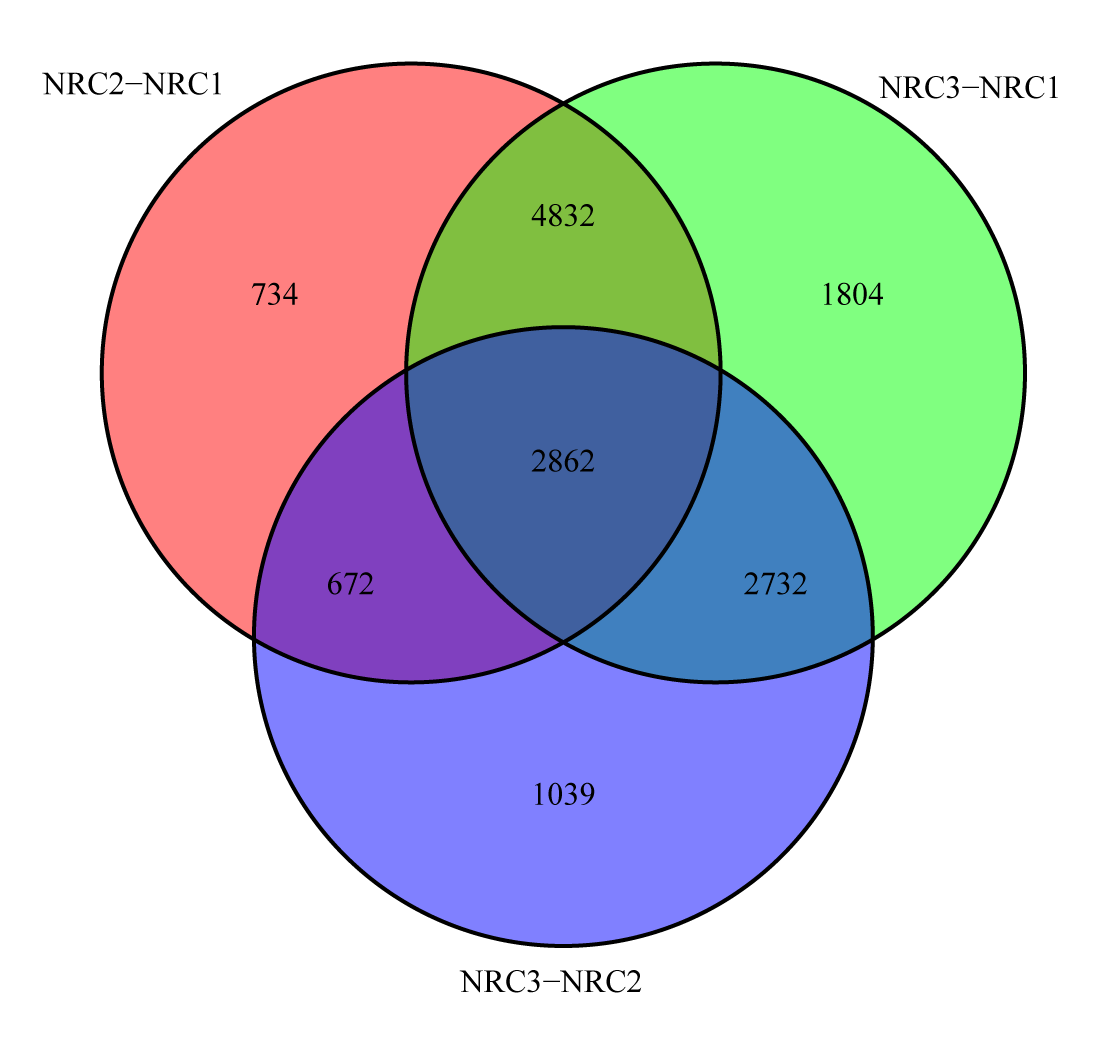

Supplement: Supplementary file 5 — Additional file 5. Figure S5. DEGs among the three necroptosis-related clusters [file 12967_2022_3431_MOESM5_ESM.tif]

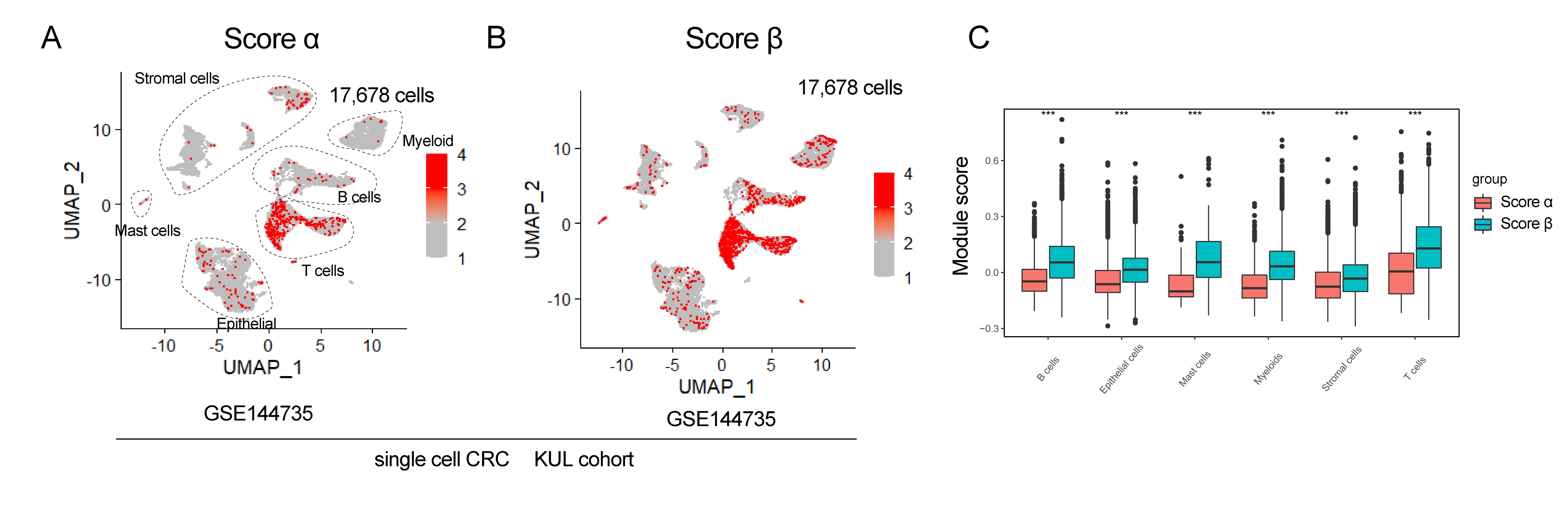

Supplement: Supplementary file 6 — Additional file 6. Figure S6. Single-cell analysis of necroptosis-based classification in CRC, related to Figure 5 (A-B) UMAP plot show score α and β in 17,678 single cells of KUL cohort. (C) Box-plot shows score of two signatures in different cell types of KUL cohort. The statistical difference of two groups was compared through the wilcox test. *P < 0.05; **P < 0.01; ***P < 0.001. [file 12967_2022_3431_MOESM6_ESM.tif]

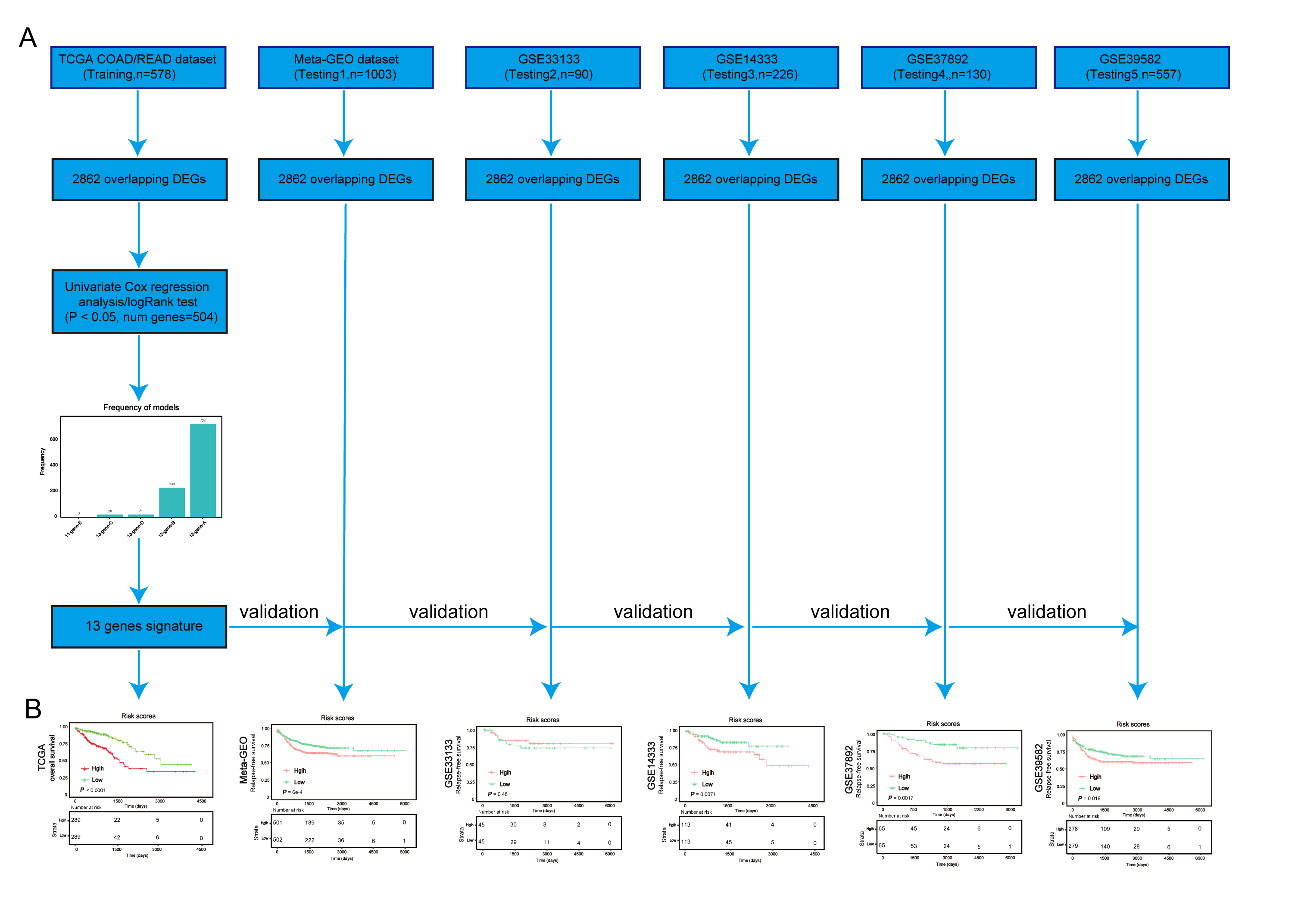

Supplement: Supplementary file 7 — Additional file 7. Figure S7. Construction and validation of the prognostic NRG_score (A-B) The workflow of construction and validation of the signature for calculating NRG risk score (NRG_score). [file 12967_2022_3431_MOESM7_ESM.tif]

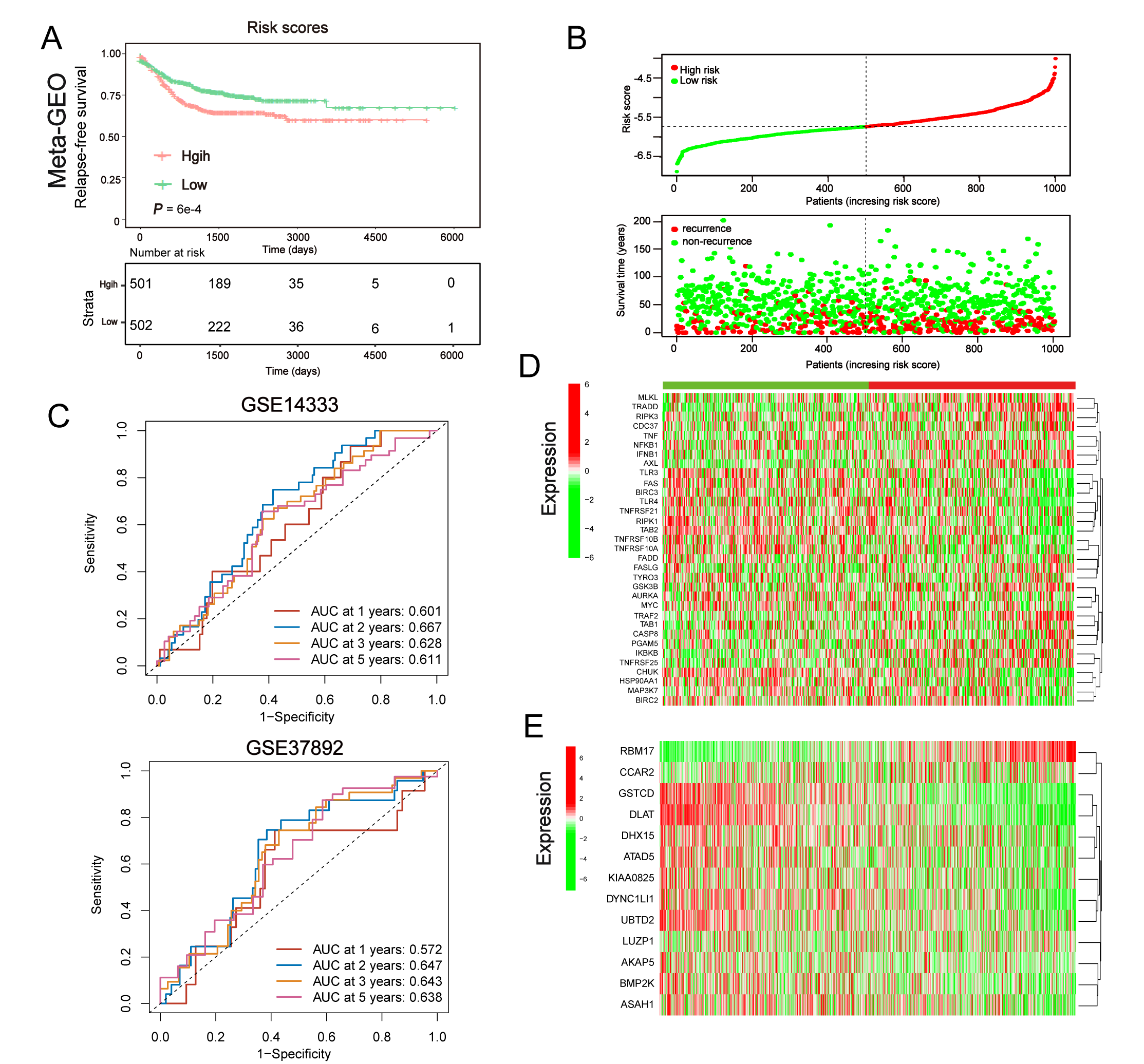

Supplement: Supplementary file 8 — Additional file 8. Figure S8. Construction and validation of the prognostic NRG_score, related to Figure 7 (A) Kaplan–Meier analysis of the survival rate between the two groups in meta-GEO cohort. (B) Ranked dot and scatter plots showing the NRG_score distribution and patient survival status. (C) ROC curves to predict the sensitivity and specificity of 1-, 2-, 3-, and 5-year survival according to the NRG_score in GSE14333 and GSE37892 cohort. (D-E) Differences in the expression of 33 NRGs and 13 genes among the twogene subtypes. [file 12967_2022_3431_MOESM8_ESM.tif]

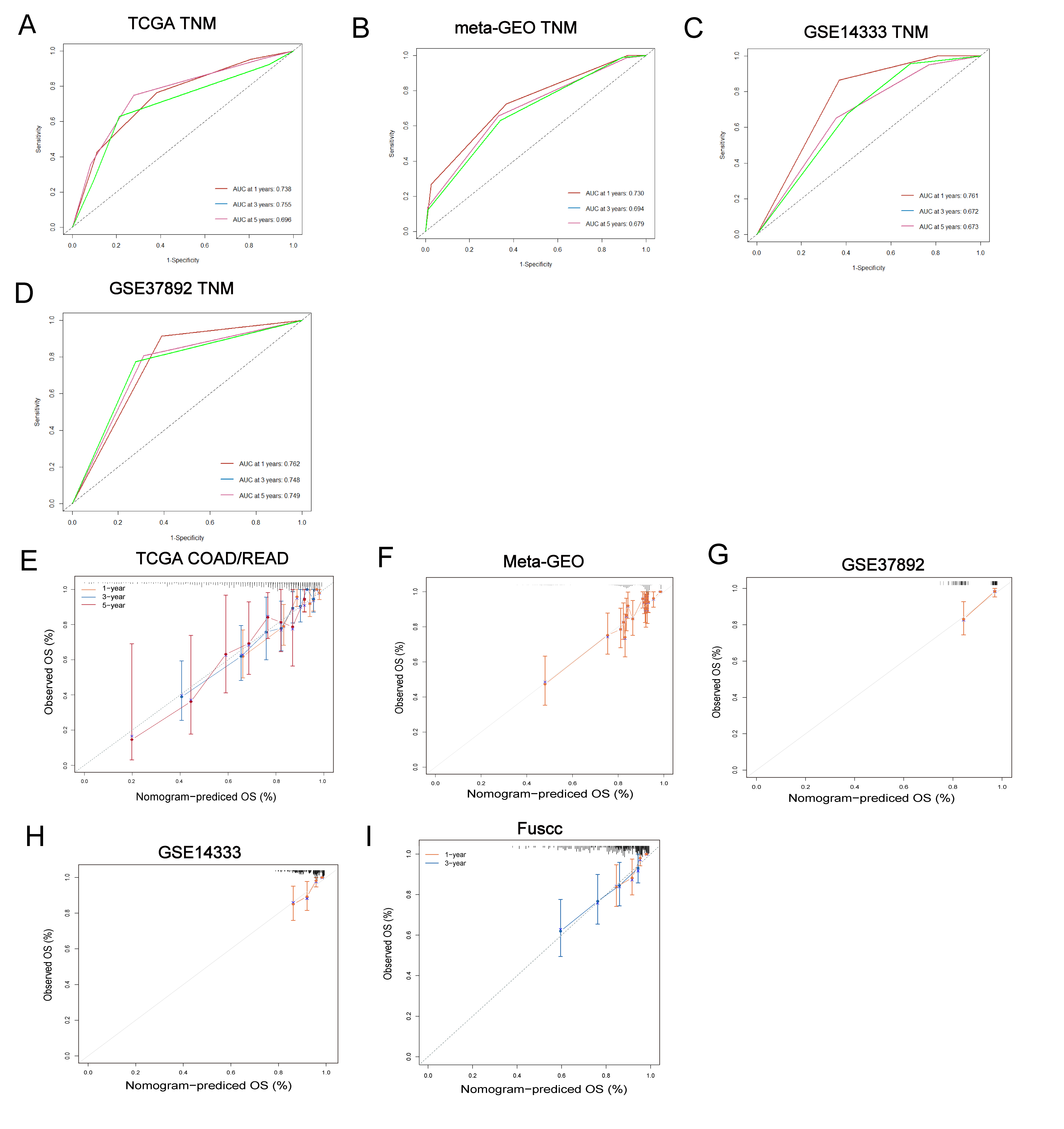

Supplement: Supplementary file 9 — Additional file 9. Figure S9. Developing a nomogram to predict patients’ survival, related to Figure 10 (A-D) ROC curves for predicting the 1-, 3- and 5-years, ROC curves in the training (TCGA), testing (meta-GEO), GSE37892, GSE14333 and FUSCC cohorts based on TNM stage systems (E-I) Calibration curves of the nomogram for predicting of 1-, 3-, and 5-year survival rate in the training, testing, GSE37892, and GSE14333 sets, and FUSCC cohort. [file 12967_2022_3431_MOESM9_ESM.tif]
